# Watching a signaling protein function: What has been learned over four decades of time-resolved studies of photoactive yellow protein

**DOI:** 10.1063/4.0000241

**Published:** 2024-04-08

**Authors:** Friedrich Schotte, Hyun Sun Cho, Fred Dyda, Philip Anfinrud

**Affiliations:** 1 National Institutes of Health, NIDDK, LCP, Bethesda, Maryland 20892, USA; 2 National Institutes of Health, NIDDK, LMB, Bethesda, Maryland 20892, USA

## Abstract

Photoactive yellow protein (PYP) is a signaling protein whose internal p-coumaric acid chromophore undergoes reversible, light-induced *trans*-to-*cis* isomerization, which triggers a sequence of structural changes that ultimately lead to a signaling state. Since its discovery nearly 40 years ago, PYP has attracted much interest and has become one of the most extensively studied proteins found in nature. The method of time-resolved crystallography, pioneered by Keith Moffat, has successfully characterized intermediates in the PYP photocycle at near atomic resolution over 12 decades of time down to the sub-picosecond time scale, allowing one to stitch together a movie and literally watch a protein as it functions. But how close to reality is this movie? To address this question, results from numerous complementary time-resolved techniques including x-ray crystallography, x-ray scattering, and spectroscopy are discussed. Emerging from spectroscopic studies is a general consensus that three time constants are required to model the excited state relaxation, with a highly strained ground-state *cis* intermediate formed in less than 2.4 ps. Persistent strain drives the sequence of structural transitions that ultimately produce the signaling state. Crystal packing forces produce a restoring force that slows somewhat the rates of interconversion between the intermediates. Moreover, the solvent composition surrounding PYP can influence the number and structures of intermediates as well as the rates at which they interconvert. When chloride is present, the PYP photocycle in a crystal closely tracks that in solution, which suggests the epic movie of the PYP photocycle is indeed based in reality.

## INTRODUCTION

Photoactive yellow protein (PYP) is a 14 kDa blue-light photoreceptor found in *Ectothiorhodospira halophila* (*E. halophila*) that, upon light activation, triggers negative phototaxis in this organism (Meyer, 1985; Sprenger *et al.*, 1993). The ability to isolate this relatively small protein in large quantities and grow large, well-ordered crystals, combined with its photochemical reversibility, has made PYP an ideal model system for biophysical studies. Accordingly, PYP has attracted the attention of a myriad of theoretical and experimental scientists, including Keith Moffat, to whom this special issue is dedicated. In this review, we chronicle advances in our understanding of what this protein does and how it does it. The intention is not to be exhaustive, but instead to highlight results across the decades that have contributed much to our understanding of PYP, with a particular focus on time-resolved studies. These efforts have produced perhaps the most complete biophysical description of a signaling protein to date, allowing one to literally watch a protein as it functions.

## THE FIRST DECADE

In 1985, Meyer characterized soluble chromophoric proteins found in *E. halophila* and noted that this bacterium produced a significant amount of a yellow-colored protein whose molecular weight was estimated by gel electrophoresis to be 15.3 kDa and whose peak absorbance at 446 nm was found to have an extinction coefficient of 48 mM^−1 ^cm^−1^ (Meyer, 1985). This protein had not yet been observed in phototrophic bacteria, and given its abundance, Meyer speculated that it may be required for survival in extremely halophilic habitats. Indeed, this organism exhibits negative phototaxis with a wavelength dependence that matches the PYP absorption spectrum, suggesting PYP functions as a repellent photoreceptor (Sprenger *et al.*, 1993). Photoexcitation of PYP triggers a reversible photocycle that generates a long-lived, blue-shifted intermediate that is thought to trigger a structural change of the protein and produce a signaling state (Meyer *et al.*, 1987). The PYP photocycle was investigated in greater detail via time-resolved absorption spectroscopy following photoexcitation with 8 ns, 446 nm pulses (Hoff *et al.*, 1994). That study involved global analysis using singular decomposition, from which the authors identified two spectroscopically distinguishable states but four time constants: a red-shifted intermediate (peak: 460–472 nm) appeared promptly and decayed biexponentially with time constants (relative amplitudes) of 0.25 ms (0.6) and 1.2 ms (0.4) to a blue-shifted intermediate (peak: 355–357 nm) that decayed back to the ground state biexponentially with time constants of 0.15 s (0.93) and 2.0 s (0.07) (Hoff *et al.*, 1994). The authors struggled to make sense of their data and stated in their Discussion section: “We have found no straightforward physical interpretation of these data.” A proper physical interpretation would have to await a later decade.

In 1995, the structure of this protein was solved by Getzoff and co-workers at 1.4 Å resolution (Borgstahl *et al.*, 1995). The chromophore was identified as a 4-hydroxycinnamyl moiety covalently bound to Cys69 by a thioester linkage and tethered to the protein by a network of hydrogen bonds (Baca *et al.*, 1994). This chromophore is also known as p-coumaric acid (pCA), the designation used in this manuscript. Moffat and co-workers subsequently demonstrated that PYP undergoes a reversible photocycle in crystals as well, making it an ideal candidate for future time-resolved crystallographic studies of PYP (Ng *et al.*, 1995). Soon after, Kort *et al.* used high performance capillary zone electrophoresis and ^1^H nuclear magnetic resonance spectroscopy to demonstrate that the photochemical basis for the PYP photocycle involves *trans*-to-*cis* photo-isomerization of the pCA vinyl double bond (Kort *et al.*, 1996).

**FIG. 1.**
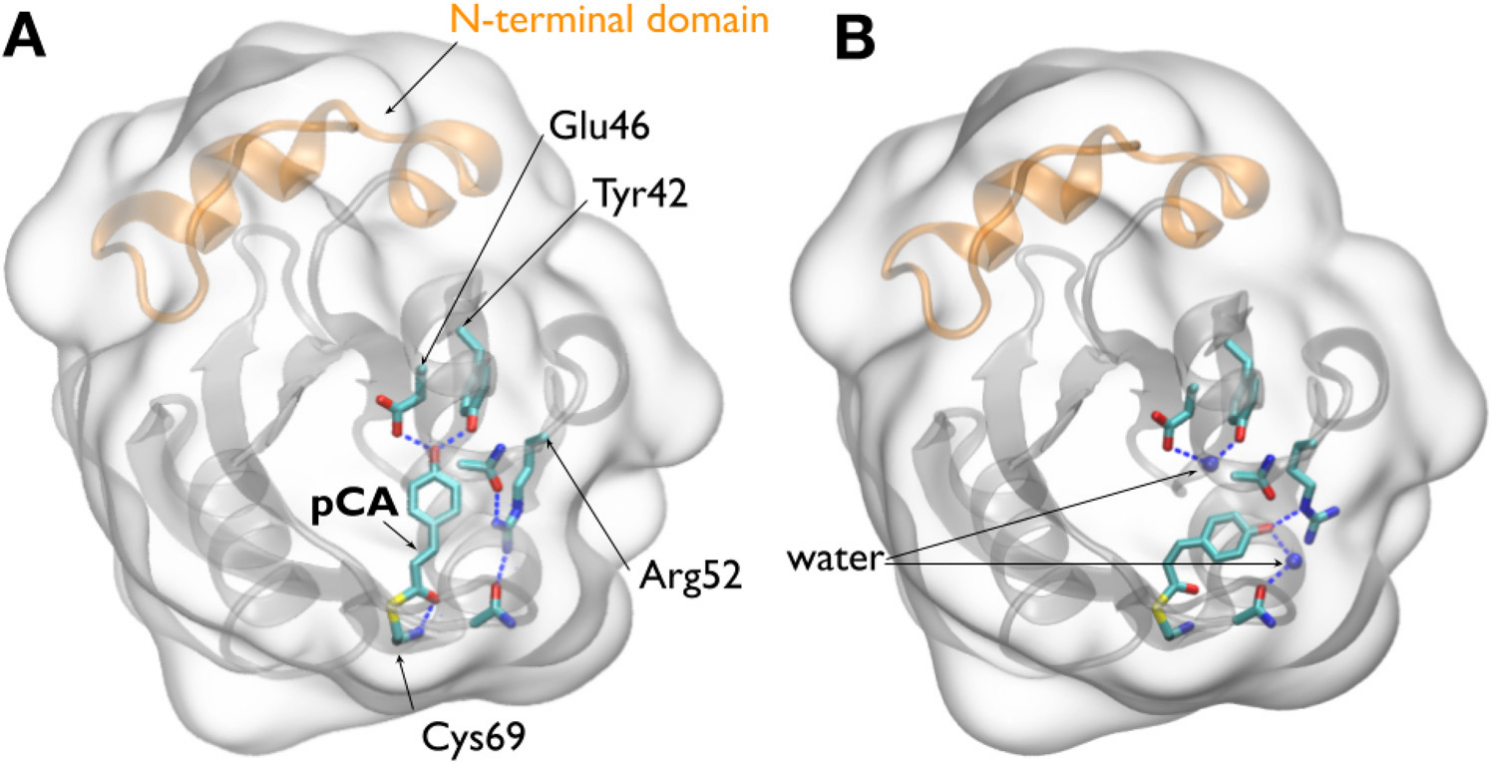
Crystal structures of PYP. (a) Structure of PYP in its pG ground state (PDB ID: 2ZOH). (b) Structure of PYP in its pB_0_ state (PDB ID: 4BBV), as determined by time-resolved Laue crystallography (Schotte *et al.*, 2012). In this state, a water molecule penetrates into the interior of the protein and hydrogen bonds to Glu46 and Tyr42. A second water molecule hydrogen bonds to the pCA phenolate oxygen, which is protonated in this state. The surfaces of both figures are rendered as glass and the backbone as ribbon. The pCA chromophore and Arg52, along with their hydrogen-bonding partners, are rendered as licorice. Dashed blue lines depict hydrogen bonds. The 25-residue N-terminal domain, colored orange, caps the β-scaffold of this sensory protein (Cho *et al.*, 2016).

For the sake of subsequent discussion, the structures shown in Fig. 1 juxtapose the ground (pG) and longest-lived intermediate (pB_0_) structures determined in later time-resolved studies (Schotte *et al.*, 2012). These structures highlight hydrogen bonding interactions with the pCA chromophore as well as relevant crystallographic water molecules. Moreover, the 25-residue N-terminal domain is highlighted, as it purportedly plays a crucial role in producing the signaling state.

## THE SECOND DECADE

**FIG. 2.**
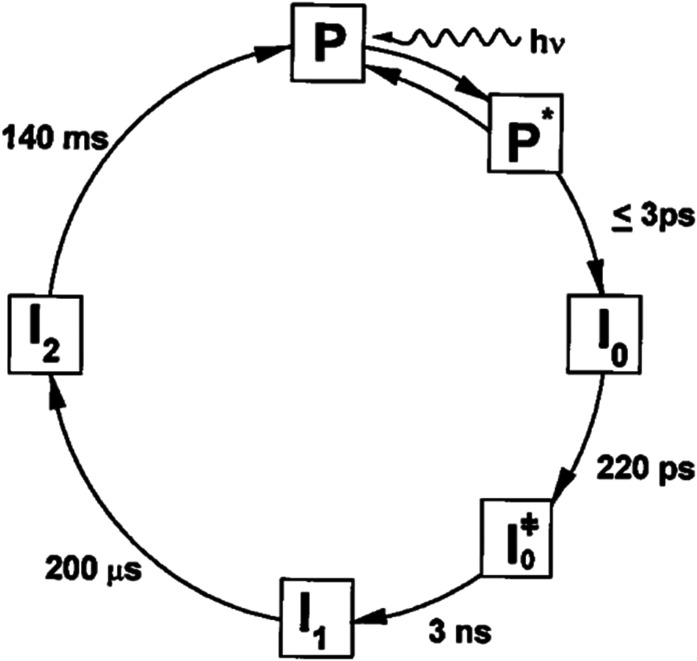
Revised photocycle for PYP from Ujj *et al.* (Ujj *et al.*, 1998). Reprinted with permission from Ujj *et al.*, Biophys. J. **75**, 406–412 (1998). Copyright 1998 Biophysical Society.

Early investigations into the reversible photocycle of PYP were conducted with nanosecond or poorer time resolution (Hoff *et al.*, 1994; Meyer *et al.*, 1987) and characterized three states: dark state (P), red-shifted intermediate (I_1_), and blue-shifted intermediate (I_2_). In 1998, Atkinson and co-workers reported a more detailed photocycle based on picosecond time-resolved spectra (Fig. 2) (Ujj *et al.*, 1998). Their time resolution of ∼5 ps was marginally sufficient to characterize stimulated emission from the excited electronic state as well as transient absorbance of early intermediates, from which they identified three additional species: PYP in its first excited electronic state (P^*^) and two early ground state intermediates (GSIs), I_0_ and I_0_^‡^. The disappearance of P^*^ and the concomitant appearance of I_0_ was too fast to be accurately characterized with 5 ps time resolution, so the authors put an upper limit of 3 ps for that step in the photocycle. This study led to the inclusion of two early ground-state intermediates that precede the previously characterized red-shifted I_1_ state, as shown in Fig. 2. We reproduce this figure not because it is the best representation of the PYP photocycle, but because it was the first to demonstrate that ultrafast processes play a significant role in launching PYP on a photocycle that leads to a signaling state. As we will soon explain, things were about to get even more complicated.

This decade also bore witness to the power of time-resolved structural studies. To kick things off, Genick *et al.* solved a time-resolved structure of the long-lived pB state of PYP with millisecond time-resolved crystallography (Genick *et al.*, 1997). Soon after, Getzoff produced two high resolution structures of PYP from a single 200 × 200 × 800 *μ*m^3^ PYP crystal chilled to 149 K (Genick *et al.*, 1998). The resolution of the first structure, acquired in the dark, was 0.82 Å and that of the second, acquired during continuous illumination with 460 nm light, was 0.85 Å. The aim was to cryogenically trap and characterize the structure of the first intermediate in the PYP photocycle, which presumably corresponds to pR. The refined occupancy of the dark state that persisted in the light-illuminated dataset was relatively high at 75%. Nevertheless, the quality of the data was sufficient to refine both dark and light structures. Because cryogenic temperatures constrain protein motion, the structural modeling and refinement was confined to the pCA chromophore and its 11 nearest neighbor amino acid residues. However, questions remained. The light structure showed a highly unfavorable −80° double bond torsion angle between C_2_=C_3_, suggesting isomerization around this double bond was incomplete, leaving the pCA chromophore in a highly strained state that may be responsible for driving the PYP photocycle. In contrast, a follow-up cryo-crystallographic study conducted at a lower temperature, 85 K, showed complete *trans*-to-*cis* isomerization of the C_2_=C_3_ bond (Kort et al., 2004). Kort *et al.* suggested that the PYP photocycle is driven not by strain in the torsion angle of the C_2_=C_3_ bond, but instead by the elevated potential energy of the *cis*-conformation in the chromophore pocket. Moreover, they suggested that the discrepancy in the cryo-trapped structures might be resolved if the higher temperature 149 K maps of Genick *et al.* were reinterpreted as a mixture of photoinduced and possibly radiation damage-induced intermediates. Soon after, Moffat and co-workers acquired the first time-resolved electron density map calculated from Laue diffraction acquired from an early intermediate in the PYP photocycle (Perman *et al.*, 1998). Using the pump-probe (PP) method, they recorded diffraction patterns generated from 150 ps duration, broadband (0.3–1.8 Å) x-ray pulses synchronized to arrive after photoactivation of small crystals (80 × 80 × 150 *μ*m^3^) of PYP with 495 nm, 7 ns FWHM laser pulses at a fluence of ∼2.3 mJ mm^−2^. The extent of photoinitiation was estimated to be 12%, and the effective pump-probe time delay was approximately 2 ± 2 ns. Despite issues with the structure refinement, the difference maps were fully consistent with a *cis* conformation of the pCA chromophore and presumably corresponds to pR, the red-shifted spectroscopic intermediate. Moffat and co-workers subsequently demonstrated the ability to acquire Laue diffraction snapshots spanning more than seven decades of time with time resolution limited only by the 7 ns FWHM laser pulse used to trigger the PYP photocycle (Ren *et al.*, 2001). While the difference electron density maps in their time series exhibited time-dependent changes, the S/N ratio of the data were poor, and the title's promise of a “Molecular Movie at 1.8 Resolution…” remained unfulfilled. Nevertheless, this seminal work made clear that the quest to generate a molecular movie of the PYP photocycle was possible, but would require improved experimental approaches.

## TIME-RESOLVED SPECTROSCOPIC STUDIES

Two decades after the discovery of PYP, numerous time-resolved spectroscopists jumped onto the PYP bandwagon and employed a variety of complementary spectroscopic methods to investigate the PYP photocycle, several of which are summarized here.

### Time-resolved fluorescence

**FIG. 3.**
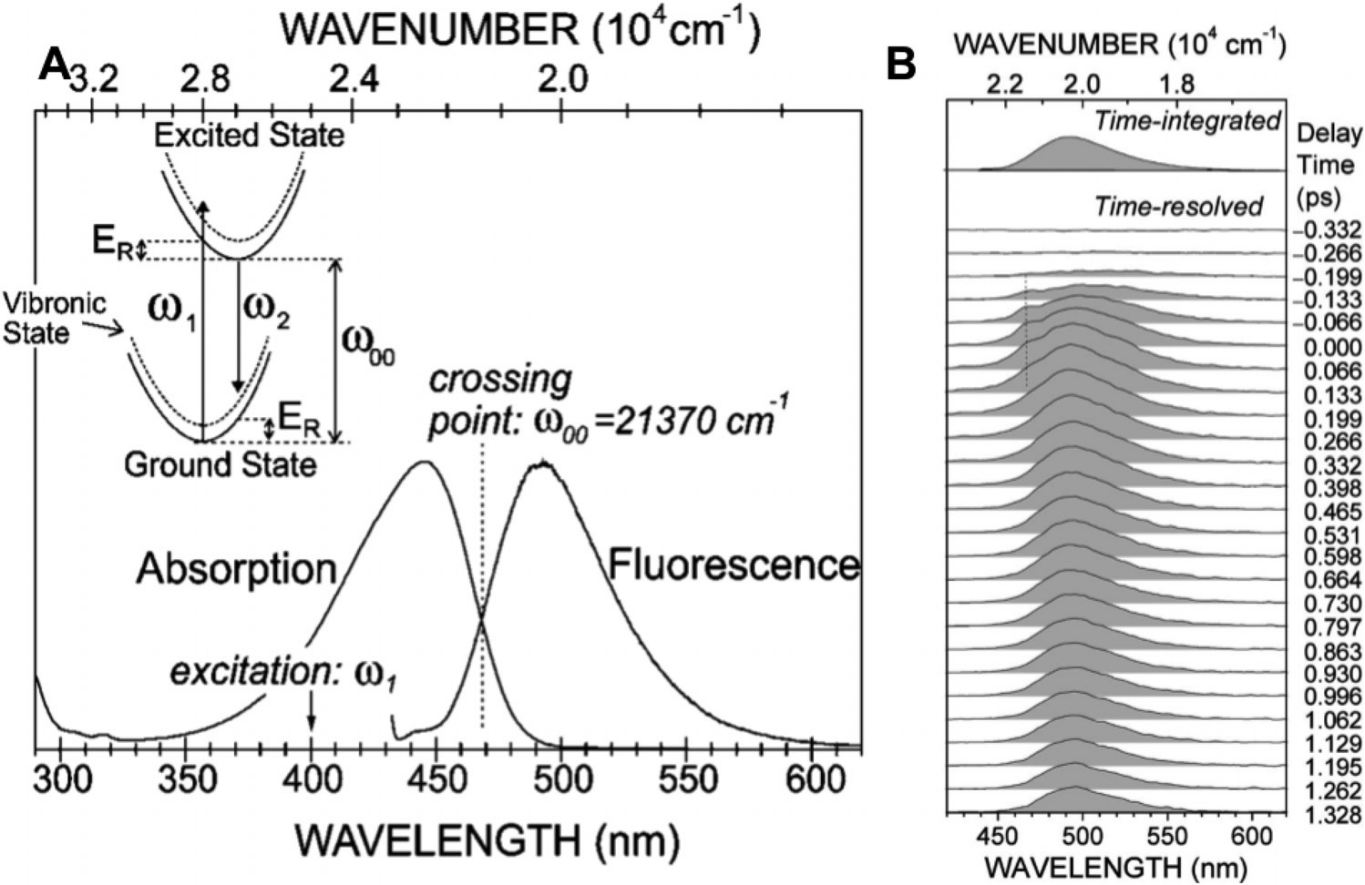
(a) Normalized stationary absorption and fluorescence spectra of PYP at room temperature. The arrow at 400 nm (25 000 cm^−1^) indicates the excitation energy for stationary and time-resolved experiments. The dotted line represents the energy ω_00_ at the crossing point of the absorption and fluorescence spectra. The schematic potential-energy diagram is illustrated in the inset with the crossing point of the absorption and fluorescence spectra denoted ω_00_, excitation energy ω_1_, emitted photon energy ω_2_, and redistribution energy E_R_. (b) Time-resolved fluorescence spectra of PYP recorded by Kerr-gate spectroscopy. A hump centered at a wavelength of 468 nm and at a delay time of 0.0 ps, indicated by a dotted line, is due to Raman scattering from water. The time-integrated fluorescence spectrum is also shown on top for comparison (Nakamura *et al.*, 2007). Adapted with permission from J. Chem. Phys. 127(21), 215102 (2007). Copyright 2007 AIP Publishing LLC.

Transient absorption spectra can provide much useful information about photo-generated early intermediates; however, transient absorption spectra include contributions from both absorbance and stimulated emission, which makes their interpretation more complex and model dependent. On the other hand, time-resolved fluorescence emission spectra report on the excited state and are essentially free of complications from transient absorbance. In 2007, Nakamura and co-workers reported time-dependent fluorescence emission spectra of PYP using the Kerr-gating method following 400 nm excitation with 180 fs time resolution (Nakamura *et al.*, 2007). Shifting their excitation wavelength to higher energy enabled them to characterize the complete fluorescence emission spectrum free of interference from the pump pulse (aside from Raman scattering). The fluorescence was found to be short-lived, decaying non-exponentially with time constants of approximately 0.4 ps (0.22) and 2.3 ps (0.78), which correspond to the average of time traces recorded at 512, 515, and 518 nm. However, its center frequency was stable from 0 to 1.3 ps, and its width narrowed significantly during the same time window. To better understand the implications of these results, refer to Fig. 3.

In the inset of Fig. 3(a), the horizontal axis of the potential energy diagram corresponds to nuclear coordinates while the vertical axis corresponds to energy. Since photon absorption (ω1) and emission (ω2) are rapid compared to nuclear motion, the absorption and emission arrows are vertical. The measured absorption spectrum shown in Fig. 3(a) is relatively broad, whose profile reflects Frank–Condon overlap between ground and excited vibronic states, and clearly favors higher vibrational states in the excited state. For similar reasons, the fluorescence emission spectrum is broad and an approximate mirror image of the absorbance spectrum with its peak offset by an amount referred to as the Stokes shift, which according to their data are 2090 cm^−1^. Since the magnitude of the PYP Stokes shift is not unusually large, the absorbance and emission spectra are near mirror images of each other, and the center frequency of the fluorescence emission spectrum is stable during the excited state lifetime, one can conclude that the equilibrium configuration of the excited electronic state is not far from that for the ground state, as illustrated in the inset potential-energy diagram in Fig. 3(a).

How might one explain rapid spectral narrowing of the fluorescence emission spectrum? Since excited vibrational states relax rapidly to their ground vibrational states in both ground and excited electronic states, the fluorescence photon energy is lower than the absorbed photon energy, which contributes to the magnitude of the Stokes shift. However, emission that occurs before vibrational energy relaxation in the excited electronic state is complete will be broadened by its elevated vibrational temperature. For example, given 25 000 cm^−1^ (400 nm) excitation and ω_00_ = 21 370 cm^−1^, the excess photon energy deposited into the chromophore upon promotion into its first excited electronic state is approximately 3630 cm^−1^, which promptly increases the vibrational temperature of the chromophore. The pCA chromophore has 18 atoms including the sulfur linkage to the protein backbone, which provides 3N - 6 = 48 vibrational degrees of freedom, each of which has on average 207 cm^−1^ vibrational energy at room temperature (298 K), or 9938 cm^−1^ in total. The sudden deposition of 3630 cm^−1^ energy into as many as 48 vibrational degrees of freedom of the PYP chromophore would jump its vibrational temperature by over 100 K. The width of the fluorescence emission spectrum is broadened by this temperature jump and narrows as the chromophore cools back to ambient temperature via intra- and inter-molecular vibrational energy redistribution. Hence, this spectral narrowing of the fluorescence emission spectrum closely tracks the chromophore temperature. As expected, the dynamics of spectral narrowing was found to be non-exponential; modeling with a biexponential function recovered time constants of 0.2 and 1.5 ps, with the shorter time constant being dominant.

Chromophores typically have an inverse radiative rate of several nanoseconds. What makes a chromophore an efficient fluorophore is when non-radiative pathways back to the ground state are far slower. The fluorescence lifetime in PYP is very short, which implies it is quenched by rapid internal conversion to the ground electronic state via pathways that lead back to the starting *trans* state or to the *cis* photoisomer. The absorbed photon energy is, therefore, converted into heat in two steps: upon photoexcitation (∼3630 cm^−1^ excess energy with 400 nm excitation), and then upon electronic relaxation back down to the ground state (>20 000 cm^−1^). Clearly, the temperature jump experienced by the pCA chromophore when transitioning back to the ground electronic state is significantly larger than that experienced upon initial photoexcitation. For example, if the energy of a 450 nm photon (22 222 cm^−1^) was thermally distributed among 48 vibrational degrees of freedom in pCA, its temperature jump would have an upper limit of 666 K. This sudden heating “rattles” the cage of atoms surrounding the chromophore in the protein, surely increasing the crystallographic B factors for the chromophore and its immediate surroundings over time scales comparable to vibrational relaxation and may also populate higher energy conformational substates with subtle differences in structure.

**FIG. 4.**
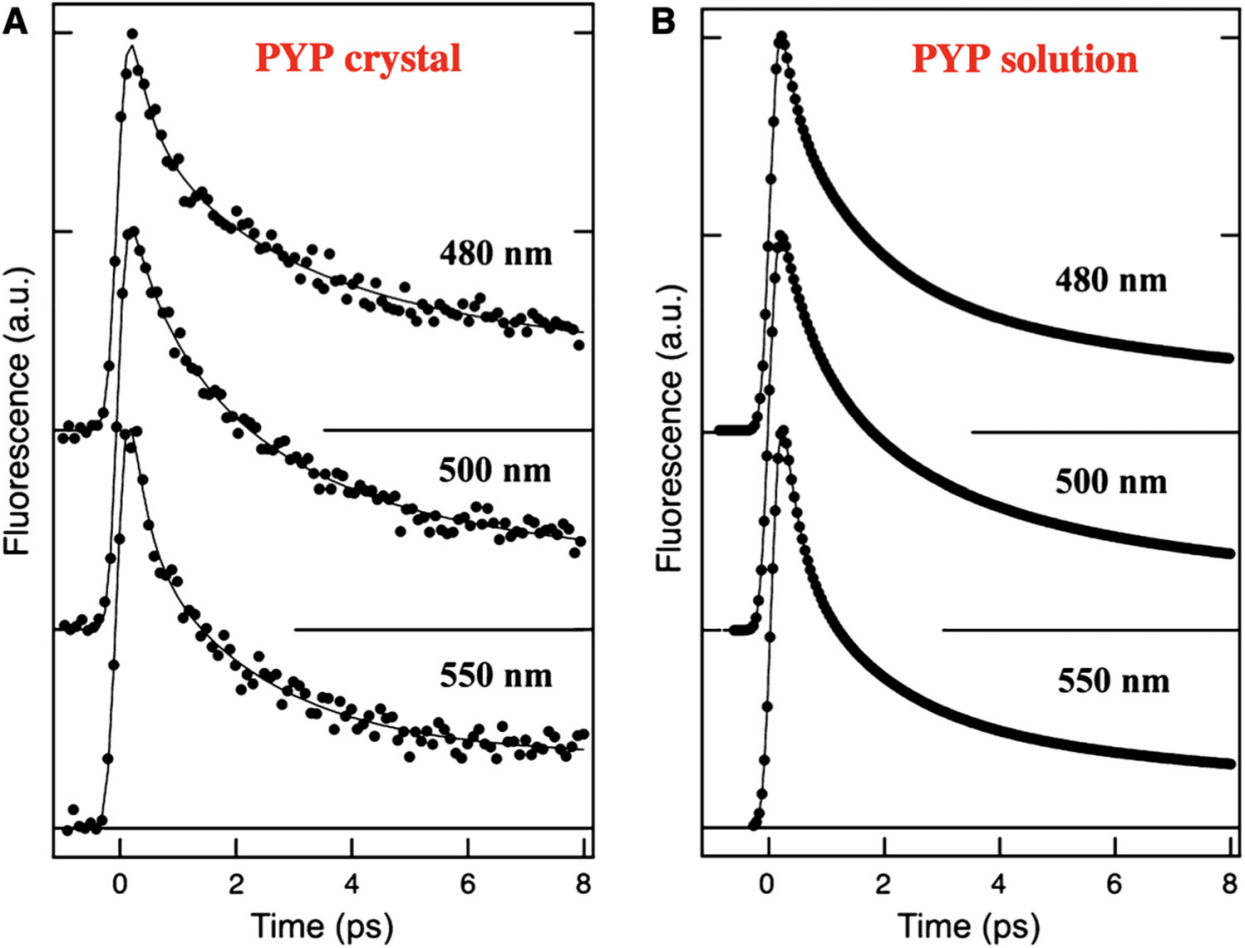
Time-resolved fluorescence emission of PYP in a crystal and in solution at three selected wavelengths following 410 nm excitation. The emission intensities were recorded with ∼200 fs time resolution using the fluorescence upconversion method (Chosrowjan *et al.*, 2015). Reprinted with permission from Chosrowjan *et al.*, FEBS J. **282**(16), 3003–3015 (2015). Copyright 2015 FEBS.

Seven years later, in 2015, Tanaka and co-workers compared ultrafast fluorescence dynamics from PYP in solution and in a crystal via a fluorescence upconversion method that achieved ∼200 fs time resolution (Chosrowjan *et al.*, 2015). They recorded the relative fluorescence intensity as a function of time for three spectral slices of the fluorescence emission spectrum after photoexcitation with a 410 nm laser pulse (Fig. 4). The solution and crystal data both required three rates to model the decay dynamics. Near the peak of the time-averaged fluorescence emission spectrum, 500 nm, the rates and relative amplitudes were reported to be 0.45 ps (0.28), 1.8 ps (0.45), 14 ps (0.27) for PYP in solution and 0.47 ps (0.18), 2.1 ps (0.54), 19 ps (0.28) for PYP in a crystal. The similarity between the solution and crystal rates is striking and suggests that the excited state dynamics are only minimally affected by the crystal packing constraints imposed on the PYP structure.

### Transient absorbance spectroscopy

**FIG. 5.**
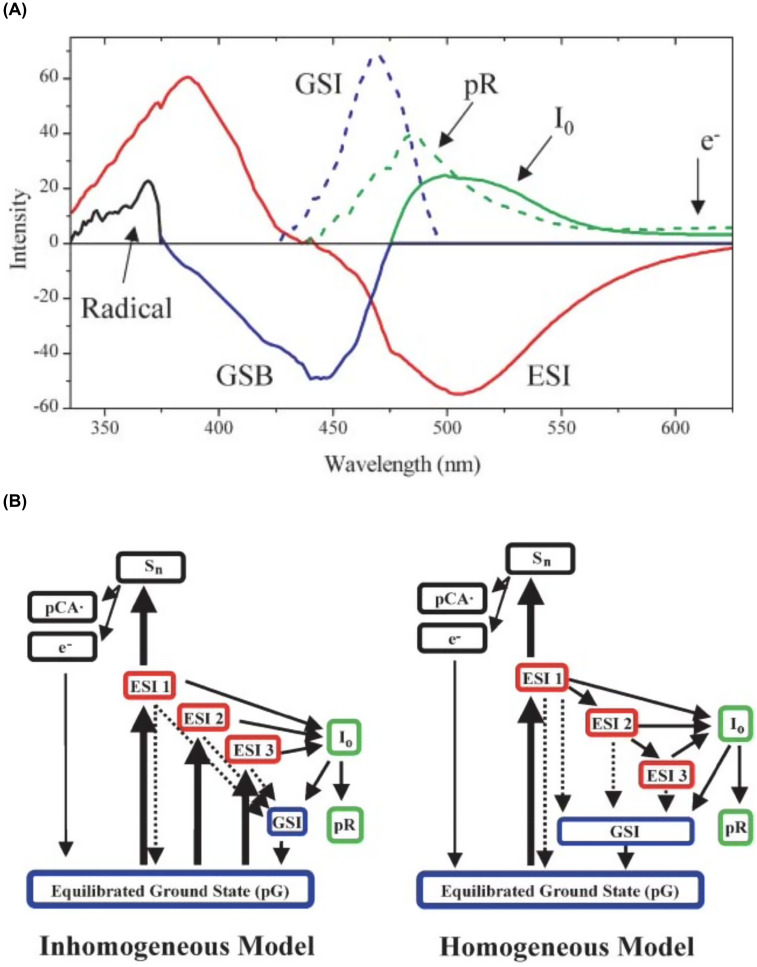
(a) Species-associated spectra (SAS) deduced from global analysis of PP (pump-probe) and PDP (pump-dump-probe) traces. The red curve is the SAS associated with the ESI1, ESI2, and ESI3 (excited state intermediate) states. The dashed blue curve is the ground-state intermediate, and the solid green and dashed green curves are the I0 and pR photoproducts, respectively. The solid blue curve is the bleach common to all transient ground states. The black curve is the pCA radical spectrum. These estimated spectra were also supported with the global fit of polarization-dependent PP data, which is published elsewhere (van Stokkum *et al.*, 2004). (b) Connectivity schemes compatible with the data and used in the global analysis: inhomogeneous model and homogeneous model. Dynamical states are separated into four classes: excited state (red), ground state (blue), photocycle products (green), and two-photon ionization dynamics (black). ESI1, ESI2, and ESI3 refer to the excited-states #1, #2, and #3, respectively. pG is the equilibrated ground-state species, and GSI is the ground-state intermediate. Thick solid arrows represent the initial excitation process from the laser pulse and thin solid arrows represent the “natural” PP population dynamics. The dashed arrows represent the population transfer dynamics that may be enhanced with the dump pulse (Larsen *et al.*, 2004). Reprinted with permission from Larsen *et al.*, Biophys. J. **87**(3), 1858–1872 (2004). Copyright 2004 Biophysical Society.

In 2004, Larsen *et al.* investigated the PYP photocycle via traditional pump-probe as well as more exotic pump-dump-probe (PDP) transient absorbance spectroscopy and generated significantly more complex models for understanding the early events in the PYP photocycle (Fig. 5) (Larsen *et al.*, 2004). Their pump-dump-probe transient absorption spectra were acquired with ∼40 fs time resolution using 395 nm excitation pulses and 505 nm dump pulses synchronized to arrive at variable delay times after the photoexcitation pulse. When the dump pulse arrives while the pCA chromophore is in its excited electronic state, it stimulates emission back to the ground electronic state, depopulating the excited state and short-circuiting the photocycle. Comparing the transient absorption dynamics with and without the dump pulse contributed to a more detailed understanding of the underlying processes in the PYP protein. It is worth noting that photoexcitation with femtosecond laser pulses at the power density required to generate high S/N transient absorption data have very high peak powers, unlike that when exposed to sunlight, which can lead to nonlinear processes such as two-photon absorption and/or excited state absorption and can trigger chromophore ionization and production of solvated electrons. Global analysis of their data recovered numerous species-associated-spectra (SAS), including states generated by nonlinear processes, and are shown in Fig. 5(a). Their proposed connectivity for these states is shown in Fig. 5(b), where both inhomogeneous and homogeneous models were found to be compatible with their data.

The decay of the excited state intermediate (ESI) was found to be multiexponential with lifetimes of 0.6 ± 0.1 ps (50%), 2.8 ± 0.3 ps (40%), and 40 ± 10 ps (10%) and provided the basis for including three distinct excited state intermediates in their connectivity models. Their inhomogeneous model assumes photoexcitation simultaneously generates three ESI states that evolve independently, and their homogeneous model assumes ESI1 alone is populated by the photoexcitation pulse, with the others populated by sequential relaxation processes. Their inhomogeneous model recovers ESI branching ratios of 40:20:1 for generating I_0_, the first *cis* state. These results imply ∼70% of the I_0_ generated originates from ESI1, the shortest lived excited state intermediate.

Larsen and co-workers (Mix *et al.*, 2018) later revisited the PYP photocycle with a modified pump-probe method, but instead of photoexciting with 395 nm laser pulses, they employed 435 and 475 nm pulses and uncovered a small but measurable pump wavelength effect on the fluorescence and ultrafast photodynamics. The excited state relaxation was again found to have three components, but with slightly different time constants and relative amplitudes than reported earlier: 1.1 (57%), 4.7 (32%), and 34 ps (11%). Moreover, the quantum yield for generating I_0_ was found to be 33% for each of the two faster rates, and 0% for the slowest rate, for a combined yield of 0.29 with the fastest rate accounting for 64% of the total. These rates differ somewhat from rates reported in their earlier work using a 395 nm photoexcitation pulse: 0.6 ± 0.1 ps (50%), 2.8 ± 0.3 ps (40%), and 40 ± 10 ps (10%), with the fastest rate accounting for 70% of the total yield of I_0_. Note that the differences between the two faster rates are outside the uncertainties reported in their earlier work. As the rates and yields extracted from transient absorption data are model dependent, perfect agreement is not to be expected.

### Ultrafast time-domain Raman spectroscopy

**FIG. 6.**
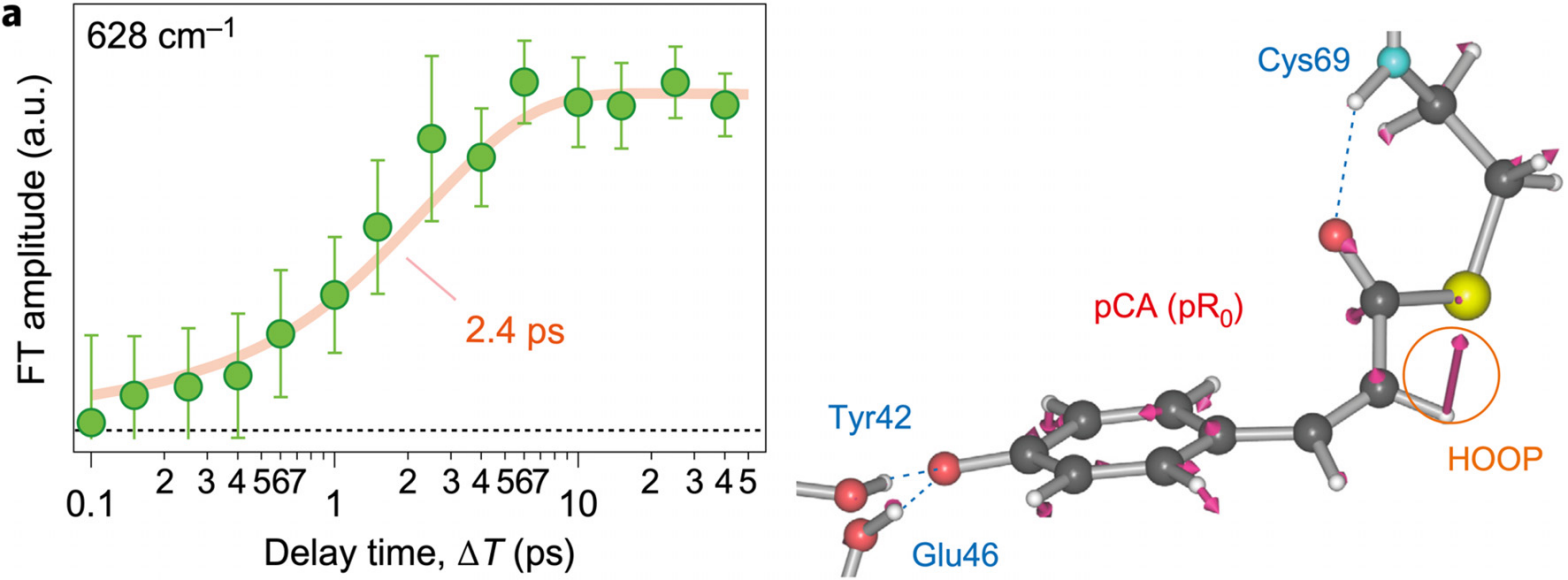
A strong Raman mode found at 628 cm^−1^ and associated with I_0_ appears with a time constant of 2.4 ps (Kuramochi *et al.*, 2017); according to density functional theory (DFT) calculations, this mode is consistent with a normal mode calculated for the pR_0_ *cis* intermediate reported by Schotte *et al.* (Schotte *et al.*, 2012). Reprinted with permission from Kuramochi *et al.*, Nat. Chem. **9**(7), 660–666 (2017). Copyright 2017 Macmillan Publishers Limited.

Tahara and co-workers (Kuramochi *et al.*, 2017) probed the early stages of the PYP photocycle with ultrafast time-domain Raman spectroscopy using sub-7 fs pulses and obtained snapshot vibrational spectra of pCA with high sensitivity. They identified a Raman band that is absent in the pG state, grows in amplitude on a similar time scale as the decay of the excited state fluorescence, and was, therefore, assigned to the first *cis* intermediate observed in the PYP photocycle (Fig. 6). Given this result, it is tempting to conclude that the transition from the excited state to a *cis* intermediate occurs with a time constant of 2.4 ps. However, this Raman band involves electronic transitions that originate from a ground electronic state in thermal equilibrium, but at this early time the pCA is definitely not in thermal equilibrium. The transition from an excited electronic state to the ground state converts a substantial fraction of the photon energy into higher vibrational modes with intra- and inter-molecular vibrational energy redistribution required to restore thermal equilibrium. Hence, the 2.4 ps appearance of the first *cis* intermediate likely reflects the time required for nascent *cis* pCA to cool back to ambient temperature and should be considered an upper limit on the time scale for formation of the *cis* isomer of pCA, which is denoted pR_0_.

### Photoisomerization potential energy surface (three-state model)

Although the excited state relaxation rates reported in different time-resolved absorption studies vary somewhat, there appears to be a general consensus that three rates are required to successfully model those dynamics. The rates recovered from the transient absorption studies are model dependent. Although prior studies typically considered both inhomogeneous and homogeneous models, the rates reported were generally based on inhomogeneous models. As discussed below, the early events in the PYP photocycle can exhibit both homogeneous and inhomogeneous behavior, so the rates and branching ratios reported in earlier studies are subject to correction.

**FIG. 7.**
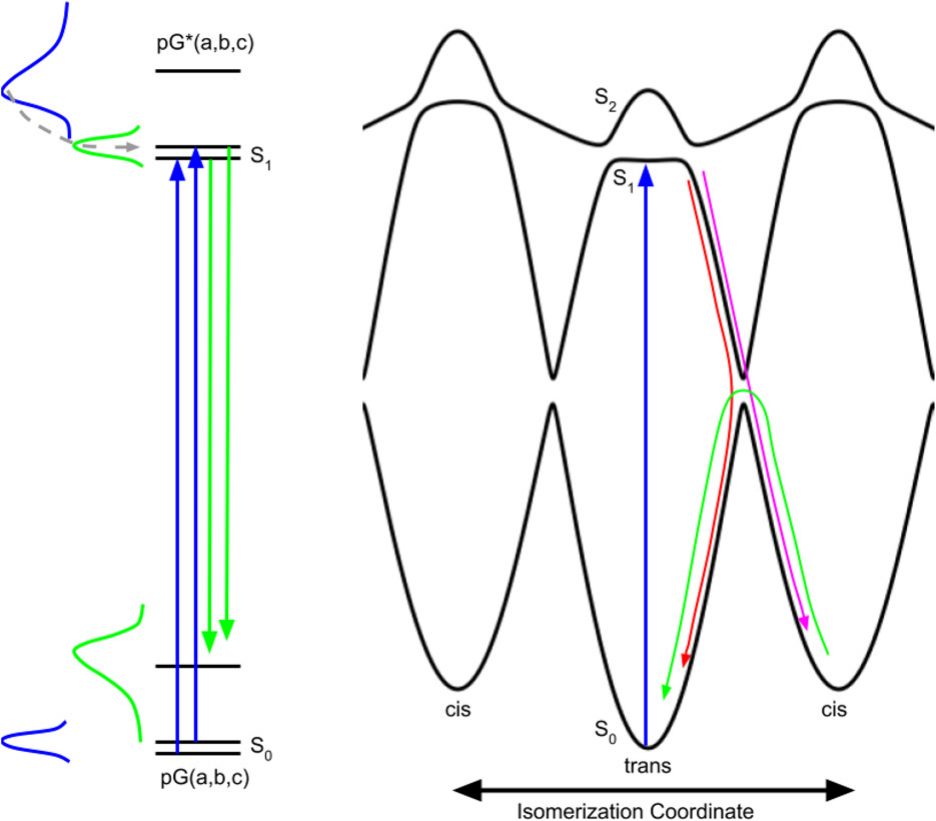
Schematic representations of PYP photoactivation and three-state potential energy curves along the isomerization coordinate. (Left) Side chain packing differences in PYP generate conformational substates (a,b,c) that coexist in thermal equilibrium with higher energy states having low occupancy (blue Gaussian). Photoexcitation of pG(a,b,c) (blue arrows) maps the ground state thermal distribution onto the first excited electronic state pG^*^(a,b,c); Frank–Condon overlap favors higher vibrational states (blue absorption spectrum), creating a non-equilibrium vibrational energy distribution that relaxes non-exponentially, first through intramolecular (sub-picosecond), then through intermolecular (ps) vibrational energy redistribution (dashed gray curve with arrow), ultimately generating an equilibrium thermal distribution in the excited state (green Gaussian). PYP fluorescence arises from radiative transitions from the excited to the ground electronic state (green arrows); Frank–Condon overlap favors higher vibrational states in the ground state (green emission spectrum). Fluorescence detected prior to vibrational energy relaxation in the excited electronic state is spectrally broadened, but rapidly narrows as the pCA chromophore cools and returns to thermal equilibrium. (Right) The torsional restraint imposed by the C_2_=C_3_ double bond in pCA leads to minima along the isomerization coordinate for *trans* and *cis* isomers, and is modeled with separate cos *θ* diabatic curves for the *trans* and *cis* states, but with *cis* exhibiting a higher potential energy due to steric effects. Photoexcitation of pCA breaks this double bond and frees this restraint, resulting in an excited state diabatic curve that is formally flat, but is modeled with a small amplitude cos θ curve due to steric effects, as in the ground electronic state. Mixing of these ground and excited diabatic curves generates avoided curve crossings with splittings that separate three diabatic curves into three adiabatic electronic states: S_0_, S_1_, and S_2_. Photoexcitation (blue arrow) from the *trans* ground electronic state (S_0_) accesses a relatively shallow minimum in the first excited electronic state (S_1_) with small barriers controlling access to the steeply sloped, reactive portion of the potential energy curve. After accessing the reactive region, a second avoided crossing is encountered and the nuclear motion either continues along the isomerization coordinate toward the *cis* minimum (magenta arrow) or is redirected back toward the *trans* minimum (red arrow), with the partitioning between these two pathways influenced by local side chain packing, i.e., conformational substates. For clarity, nuclear motion on the reactive curve is indicated only on the right side, but can also occur on the left side, with partitioning between the two directions influenced by conformational substates. Being higher in free energy than *trans*, the *cis* isomer spontaneously reverts back to the *trans* isomer, but does so on a much longer time scale that is dictated by the activation barrier at the avoided crossing that separates them (green arrow).

A three-state model for understanding photoisomerization, which is analogous to a model proposed for bacteriorhodopsin (Gai *et al.*, 1997), is shown in Fig. 7 and represents a two-dimensional slice from a multi-dimensional potential energy surface. The PYP photocycle begins with photoexcitation from the *trans* ground electronic state to the shallow minimum in the S1 state. After overcoming a barrier on either side of the minimum, nuclear motion proceeds along the steeply sloped “reactive” portion of the excited state potential and upon encountering the next avoided crossing, which corresponds to a conical intersection in multidimensional space, either continues onward to the *cis* conformation or is redirected back to the *trans* conformation. The branching ratio dictates the quantum efficiency for generating the *cis* conformation and is influenced by local structure of the side chains surrounding the pCA chromophore at the time the avoided crossing is approached. Due to steric effects, the *cis* state is higher in free energy than the *trans* state, and eventually relaxes back to the lower energy *trans* state, but on a much slower time scale that is dictated by the activation barrier for *cis* → *trans* isomerization.

The native folded structure of a protein is typically represented by atoms in fixed positions; however, thermal motion samples a range of similar structures that are similar in free energy and interconvert on a time scale dictated by the activation barriers that separate them. These so-called conformational substates, a concept introduced by Fraunfelder (Frauenfelder *et al.*, 1988), are represented by multiple energy levels in the ground and excited states on the left side of Fig. 7. The symmetry represented in the three-state model in Fig. 7 is broken by subtle structural differences associated with conformational substates, which can elevate or lower the barriers on either side of the shallow minimum (modulation of barriers not shown). These barriers not only dictate the fluorescence lifetime for each conformational substate but can also steer nuclear motion along a clockwise or counterclockwise direction. Hence, conformational substates provide the rationale for employing an inhomogeneous model when extracting kinetic rates from transient absorption spectra. However, a case can also be made for a homogeneous model. The excess vibrational energy deposited in pCA via photoexcitation elevates its temperature and accelerates the rate at which nearby barriers are surmounted, but as the temperature cools due to vibrational energy relaxation, the rate of the crossing slows. Hence, due to ultrafast thermal relaxation, one would expect the fluorescence emission to decay non-exponentially, as is observed. Larsen *et al.* (Larsen *et al.*, 2004) assigned the three lifetimes found for excited state relaxation to three excited state intermediates; an alternative and perhaps more appropriate assignment might involve only two excited state intermediates. For example, the 0.5 and 2.5 ps time constants of comparable amplitude from their earlier work could be associated with one conformational substate whose non-exponential relaxation arises from vibrational cooling that occurs on a time scale faster than the excited state lifetime (homogeneous model). The long-lived, small amplitude decay might be associated with a second conformational substate (inhomogeneous model) for which the barriers on either side of the minimum are elevated. Note that the slowest of the rates is approximately tenfold slower than the middle rate, which would require elevating the energy of the lowest nearby barrier by about 5.7 kJ mol^−1^, which is equivalent to a weak hydrogen bond.

When transitioning from S_1_ to S_0_, a large fraction of the photon energy is converted into vibrational energy in the pCA chromophore, creating a very large temperature jump that can lead to population of higher energy conformational substates, which are the likely origin of the “ground state intermediate” that is commonly invoked when extracting species associated spectra from femtosecond time-resolved transient absorption spectra (Larsen *et al.*, 2004). Clearly, a “hot” ground state is generated when transitioning from the excited state to either pG or to I_0_, but the hot I_0_ state is generally ignored when modeling the transient absorption spectra.

## PICOSECOND TIME-RESOLVED X-RAY STUDIES

### Time-resolved Laue crystallography

**FIG. 8.**
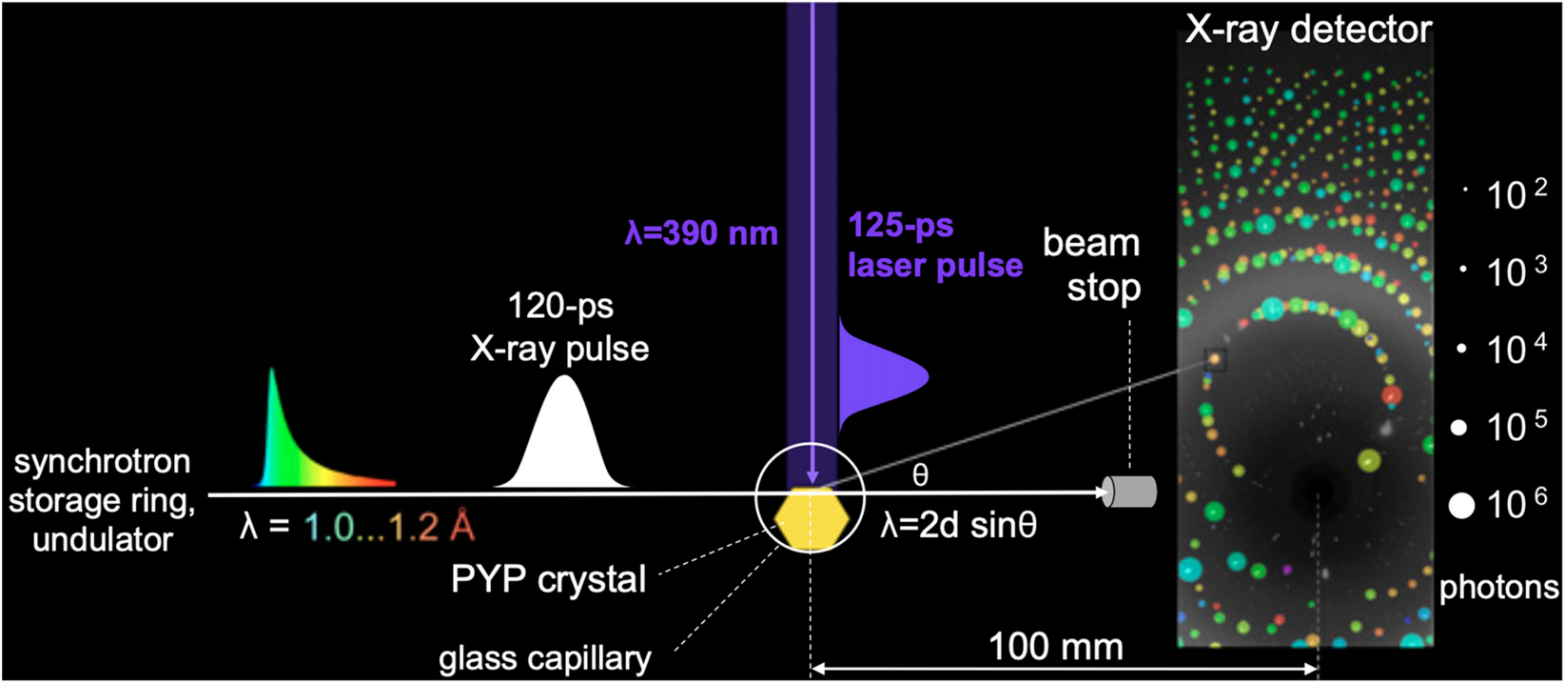
Pump-probe geometry used to acquire time-resolved diffraction snapshots. The PYP crystal is sealed in a thin-walled glass capillary. Because the laser penetration depth in PYP is shallow, an orthogonal pump-probe geometry is used in which the top edge of the protein crystal is positioned at the top edge of the focused x-ray pulse. This geometry ensures optimal overlap between the laser and x-ray illuminated volumes of the crystal. The protein crystal acts as a monochromator with various line spacings (d) and diffracts different x-ray colors (λ) in different directions (θ) according to Bragg's law (λ = 2d sin θ). Approximately 3000 spots are found in each time-resolved diffraction image. The spots in this figure are annotated according to integrated photons (spot dimension) and x-ray wavelength (spot color) (Schotte *et al.*, 2012).

The quality of time-resolved Laue diffraction data acquired in early studies was insufficient to realize the vision of a molecular movie. For example, the laser penetration depth was shallow compared to the size of the crystals used, which meant most of the diffraction intensity arose from proteins that remained in the *trans* pG state. The use of broadband x-ray radiation (0.3–1.8 Å) generated from combined wiggler and undulator insertion devices was intended to increase the number of diffraction spots in each time-resolved image (Fig. 8) and thereby increase data redundancy, but at the same time produced an abundance of harmonic overlaps that hindered the ability to accurately scale the data, and elevated the background which lowered the S/N ratio of the data. Moreover, it proved quite challenging to accurately scale and merge diffraction data acquired at different pump-probe time delays from different crystals. Since photoactivation was achieved with a laser whose pulse duration was long compared to the ∼100 ps duration of the x-ray pulses generated at synchrotrons, the time resolution achievable was limited by the nanosecond laser pulses. To mitigate these issues, an improved data acquisition strategy was developed.

**FIG. 9.**
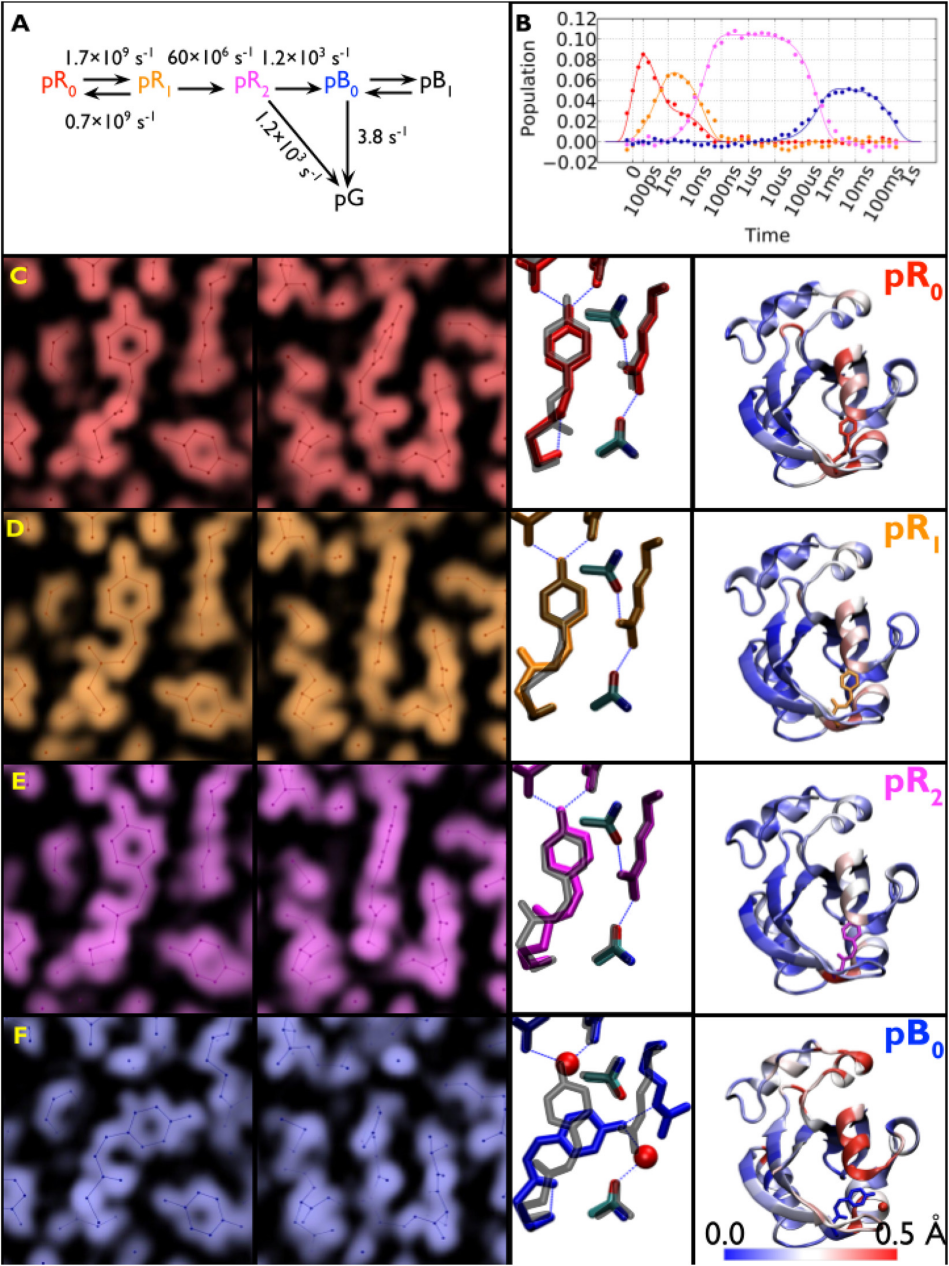
Time-resolved population of transient intermediates and their structures. (a) Kinetic model used to account for structural changes spanning ten decades; the arrows are labeled with globally refined rate constants. Half the population short-circuits to the ground state during the pR_2_ → pB_0_ transition. (b) Time-dependent populations of each intermediate in the PYP photocycle: theoretical population predicted by the kinetic model (solid lines) and least squares contributions from the four electron-density base maps (filled circles). (c)–(f) Structures for pR_0_, pR_1_, pR_2_, and pB_0_ intermediates. (Left and Center Left) Electron-density base maps were derived from global analysis and phased with refined structures (front and side views). (Center Right) Refined structures of pCA intermediates and their hydrogen-bonding partners. To highlight the structural changes leading to the corresponding intermediate, they are overlaid with a semitransparent structure (gray) of the preceding state. (Right) Color-coded rendering of the protein backbone according to Cα displacement relative to pG, as indicated by the scale (rendered with VMD) (Schotte *et al.*, 2012).

Large crystals of PYP were grown using a crystallization protocol developed to produce crystals suitable for neutron diffraction studies (Yamaguchi *et al.*, 2007), which provided sufficient volume and surface area to acquire from each crystal a complete time series over ten decades of time with four time points per decade plus numerous interleaved negative time point controls that were used to generate accurate time-dependent scattering differences (see Fig. 8). With the picosecond laser tuned to 390 nm, which is near the blue edge of the PYP absorption spectrum, its 1/e penetration depth is about 28 *μ*m, which is near the 40 *μ*m vertical dimension of the focused x-ray beam. To maximize the degree of photoactivation, an edge-finding algorithm was developed which allowed accurate placement of the crystal to ensure the focused x-ray beam passes through the portion of the crystal that experiences the highest level of photoactivation. The laser pulse was stretched to 125 ps, which is comparable to the x-ray pulse duration and sufficiently long to significantly reduce the risk of nonlinear absorption and/or multiphoton absorption, either of which can be detrimental to PYP. Moreover, photoactivation with a laser pulse that is long compared to the excited state lifetime provides multiple opportunities to trigger the PYP photocycle and help mitigate the relatively low quantum efficiency for *trans* → *cis* isomerization. Because P6 crystals of PYP are dichroic, the laser polarization was made circular to facilitate photoactivation of pCA oriented in different directions relative to the crystal axes. This improved experimental protocol enhanced the yield of the first intermediate in the PYP photocycle, which was estimated to be 10% with a laser power density of 3.5 mJ mm^−2^, compared to about 5% achieved in other studies. Instead of a broadband “white” x-ray beam, an undulator-generated “pink” beam was used whose bandwidth was sufficiently narrow to avoid harmonic overlaps, but sufficiently broad to achieve a high level of completeness with relatively few crystal orientations. Time-resolved diffraction differences from nine large crystals were scaled and merged to generate a high completeness, high S/N time-resolved dataset, from which accurate structures of four intermediates observed in the PYP photocycle and their time-dependent populations could be extracted. These four intermediates are color coded and rendered in complementary ways in Fig. 9: zoomed-in electron density maps; zoomed-in ball and stick models with hydrogen bonds highlighted; and color-coded maps of protein backbone displacement. With these rendering approaches, changes in the structure and hydrogen bonding interactions for each transition in the PYP photocycle can be visualized both locally and globally. For example, the refined pR_0_ structure depicts a highly contorted pCA chromophore with three intact hydrogen bonds between pCA and surrounding side chains, and whose structure was found to be in excellent agreement with its DFT energy-minimized structure (Schotte *et al.*, 2012). The three other structures were likewise validated by DFT. From this dataset, movies have been created that unveil the structural dynamics of PYP in real time and allow one to literally watch a protein as it functions (Schotte *et al.*, 2012) (supplementary material).

### Time-resolved small and wide angle x-ray scattering (SAXS/WAXS)

**FIG. 10.**
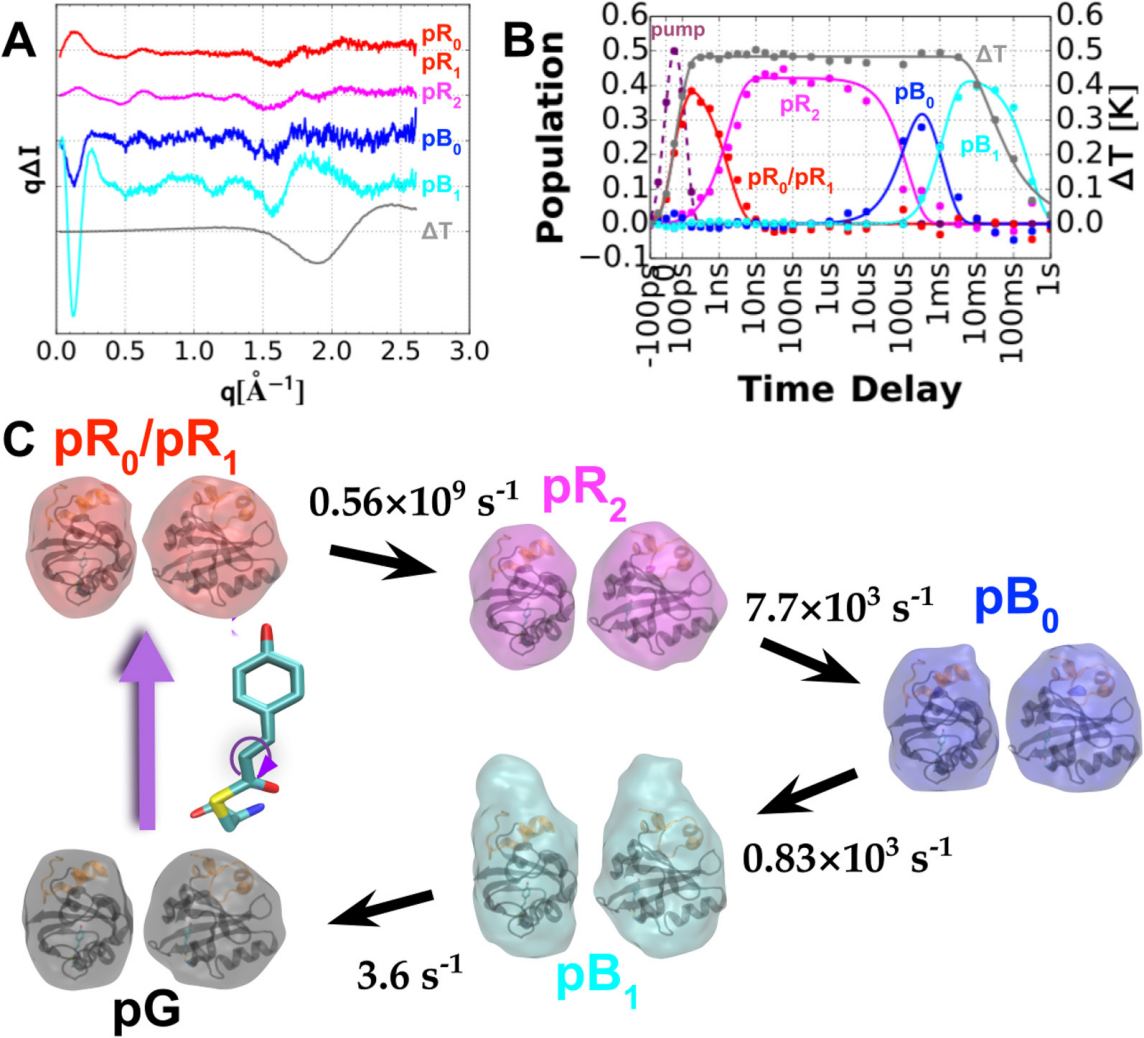
Time-resolved SAXS/WAXS scattering of PYP in solution. (a) Global analysis of time-dependent scattering differences recorded over ten decades of time spanning 100 ps to 1 s unveils four PYP intermediates plus a vector that corresponds to water differences following a 1 °C temperature jump. (b) Time-dependent amplitudes of scattering vectors shown in (a). (c) GASBOR reconstruction of particle shapes for pG and the four photocycle intermediate states found in solution along with their rates of interconversion in the PYP photocycle. The intermediates follow the color coding used in the Schotte photocycle (Cho *et al.*, 2016).

As we have shown, time-resolved Laue crystallography has provided a means to generate molecular movies that allow one to literally watch a protein as it functions. However, this capability begs the question: to what extent might crystallization of PYP affect its structure and kinetics? To address this question, Cho *et al.* (Cho *et al.*, 2016) employed the method of time-resolved small- and wide-angle x-ray scattering (SAXS/WAXS) to probe structural changes in PYP while in solution, the results of which are shown in Fig. 10. Although the data acquired is one-dimensional (when averaging x-ray scattering over its azimuthal direction), it does contain useful structural information: global changes to the protein size and shape influence the SAXS region, while local structural changes influence the WAXS region. Global analysis of the time-resolved scattering data unveiled four intermediate states denoted pR_0_/pR_1_, pR_2_, pB_0_, and pB_1_, their rates of interconversion, plus a water signal arising from the 1 °C temperature jump that accompanies photoactivation. The data were acquired following photoexcitation with 390 nm, ∼3 mJ mm^−2^ laser pulses, which were stretched to 100 ps to avoid multiphoton absorption. The pR_0_ ⇌ pR_1_ equilibrium observed in a crystal is not resolved in solution. Hence, the first intermediate observed in solution is presumed to be a mixture of these two states and is, therefore, denoted pR_0_/pR_1_. The pR_2_ → pB_0_ transition, which occurs on a submillisecond time scale, exhibits little change in the global conformation of the protein. In contrast, the pB_0_ → pB_1_ transition, which occurs within a few ms, is accompanied by a dramatic change in the protein shape that increases its radius of gyration, R_g_, from 14.7 to 16.6 Å. It is this transition that produces the PYP signaling state. The shape of the signaling pB_1_ state entails significant elongation of the long axis of the protein and unfolding of the N-terminal domain, exposing the β-scaffold to a potential binding partner. Since a structural change of this magnitude cannot occur in the crystal, its photocycle advances only as far as pB_0_. As we will soon show, the PYP photocycle in the crystal is very similar to that observed in solution, with minor differences readily rationalized in terms of structural constraints and restoring forces imposed by the crystal.

### PYP photocycle in crystal vs solution: Similarities and differences

**FIG. 11.**
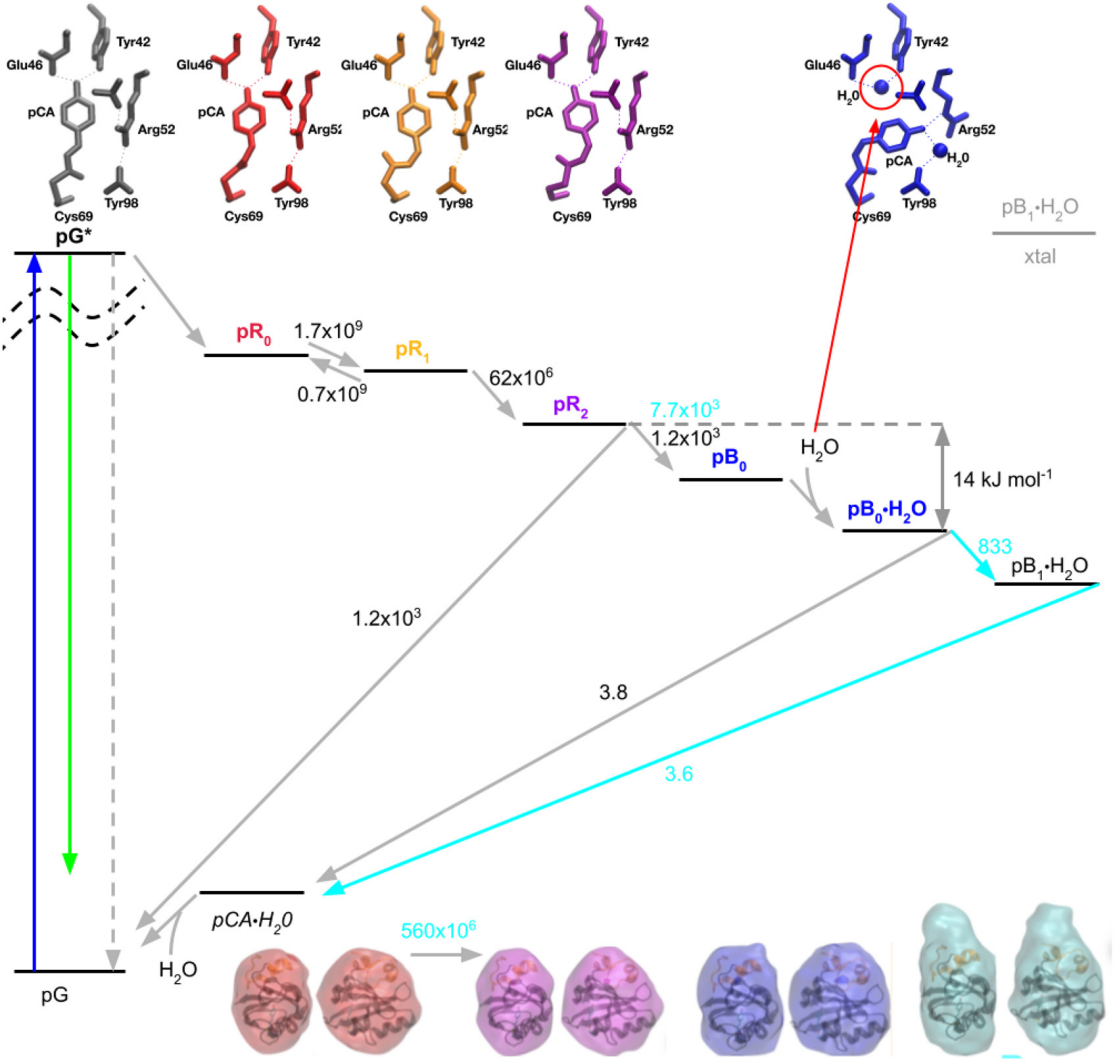
PYP photocycle with rates from both time-resolved crystallography and time-resolved x-ray scattering. Photoexcitation of pG (blue arrow) generates an electronically excited state, pG*, which returns to the ground electronic state via fluorescence (green arrow), non-radiatively back to pG (gray dashed arrow), or via a photocycle that commences with the pR_0_ state (gray arrow). The quantum efficiency for generating pR_0_ is low. The formation of pR_0_ triggers a sequence of structural changes that leads to the pB_0_ state, into which a water molecule is rapidly incorporated to generate the pB_0_•H_2_O state. The pB_0_•H_2_O → pB_1_•H_2_O transition involves partial unfolding of the N-terminal domain (orange helices that cap the top of PYP in the GASBOR envelopes; bottom) and cannot occur in the crystal. There is no structural evidence for the pB_0_ state without water nor the *trans* pCA•H_2_O state, but they are included in the photocycle based on mechanistic arguments (see the text). The kinetic rates are in units of s^−1^, with cyan values corresponding to rates extracted from solution scattering data.

A PYP photocycle based upon picosecond time-resolved x-ray structures that have been validated by density functional theory (Schotte *et al.*, 2012) is shown in Fig. 11 along with experimental interconversion rates determined at 288 K. The key structural difference between pG and all other states is pCA is in its *trans* conformation in pG vs *cis* conformation in all other states. The key structural difference between the pR and pB states is the pCA phenolate being hydrogen bonded to Tyr42 and Glu46 in all but the pB states, where it is hydrogen bonded to Arg52. The pCA carbonyl is hydrogen bonded to Cys69 in all but the pR_1_ and pR_2_ structures and is highly strained in the pR_0_ structure. The key structural difference between pR_1_ and pR_2_ is the dihedral stereochemistry of pCA between its phenolate and sulfur, which transitions from syn-syn to anti-anti. Hence, the PYP photocycle, from pR_0_ to pB_0_, is driven by strain with transient intermediates along the way stabilized by different combinations of hydrogen bonding interactions.

The formation of the first *cis* intermediate, pR_0_, shortens the overall length of the pCA chromophore and tugs on the Tyr42/Glu46 residues to which the phenolate forms very strong hydrogen bonds (Yamaguchi *et al.*, 2009), pulling these side chains downward. This step in the photocycle compacts the protein along its long axis (Cho *et al.*, 2013; Schotte *et al.*, 2012), creating strain that is partially relieved in subsequent steps in the photocycle.

The observation that pR_0_ and pR_1_ coexist in thermal equilibrium with pR_1_ being favored implies that the strain energy released by breaking the pCA carbonyl hydrogen bond with Cys69 is slightly greater than the free energy of that hydrogen bond, as has been confirmed by DFT calculations (Schotte *et al.*, 2012).

The pR_2_ → pB_0_ transition competes with a short circuited path back to pG and attains the pB_0_ state only half of the time that transition is attempted. According to transition state theory, the reaction rate is determined by temperature T and ΔG^‡^, the relative change in free energy at the transition state, and is approximated by

$$k\left(T\right)\;=\;\left(\text{RT}\right)/\left(N_{A}h\right)\;\;\text{exp}\left(-\Delta G^{‡}/\text{RT}\right),$$

where R is the gas constant, N_A_ is Avagadro's number, and h is Planck's constant. For the short circuited path, k is 1200 s^−1^ at 288 K, which implies an activation barrier of 54 kJ mol^−1^.

The pB_0_•H_2_O structure includes a water molecule hydrogen bonded to both Tyr42 and Glu46, which stabilizes this structure and slows its return to pG. The short-circuited pR_2_ → pG pathway proceeds at a rate that is approximately 315 times faster than the pB_0_•H_2_O → pG pathway, which according to transition state theory, implies a difference in their respective activation free energies of ΔΔG^‡^ = RT ln(315) = 14 kJ mol^−1^. This free energy difference is likely dominated by strain energy that persists in the pR_2_ state. This strain energy not only provides the driving force needed to break the strong hydrogen bonds between pCA and Tyr42/Glu46 but also reduces the activation energy required for thermally driven *cis* → *trans* isomerization from pR_2_ back to pG.

Mechanistically, one would expect the water molecule to access and bind to Tyr42/Glu46 after the phenolate oxygen of pCA forms a hydrogen bond with Arg52. Accordingly, the photocycle in Fig. 11 includes both pB_0_ and pB_0_•H_2_O states. Under the experimental conditions employed by Schotte *et al.*, all time-resolved difference electron density maps in which pB_0_ had a non-negligible population were best fit with an intermediate that included a water molecule hydrogen bonded to Tyr42/Glu46, which implies the rate of water penetration is fast compared to the pR_2_ → pB_0_ transition.

Since the energy required to break the *cis* double bond in pB_0_•H_2_O is high compared to hydrogen bonding interactions, one would expect thermally driven *cis* → *trans* isomerization from this state to occur prior to displacement of water hydrogen bonded to Tyr42/Glu46, which implies the photocycle passes through the *trans* pCA•H_2_O intermediate en route to pG(a,b,c). Although there is no structural evidence for a *trans* pCA•H_2_O intermediate, based on mechanistic grounds, it is included in the photocycle.

The kinetic rates in solution, as determined from a time-resolved SAXS/WAXS study, are shown in cyan (acquired at 295 K, as opposed to 288 K for the crystal study). It is instructive to consider how the crystal might influence the measured rates of interconversion. Since the quantum efficiency for generating pR_0_ in a crystal is low, PYP molecules successfully launched on a photocycle are surrounded by neighbors locked in their pG conformation, to which they are tethered through crystal contacts. This straightjacket not only constrains the spatial extent of protein motion as it progresses through its photocycle but also provides a restoring force that is biased toward pG. Hence, the interconversion rates in solution, where there is no such restraint or bias, would be expected to proceed more rapidly than in the crystal, except for the final ground state recovery step. Indeed, the putative formation of pR_2_ in the time-resolved SAXS/WAXS study, as well as in transient absorption studies, is approximately 9 times faster than the pR_1_ → pR_2_ transition found in the crystal, which implies crystal contacts elevate the activation barrier for this structural transition by 5.5 kJ mol^−1^. This free energy difference is comparable to a weak hydrogen bond, but most likely arises from restoring forces generated via crystal contacts.

The pR_2_ → pB_0_ transition in solution is 6.4 times faster than in the crystal, which implies the activation barrier for this structural transition in the crystal is elevated by 4.6 kJ mol^−1^. Being faster in solution than in a crystal, the pR_2_ → pB_0_ transition outcompetes the pR_2_ → pG pathway observed in a crystal, thereby boosting the quantum efficiency for forming pB_0_ in solution up to twofold. When free of the spatial constraints and restoring forces induced by the crystal, pB_0_ transitions to pB_1_, in which the 25-residue N-terminal domain becomes disordered and increases significantly the Rg of this state, as found in a time-resolved SAXS/WAXS study of PYP (Cho *et al.*, 2016). This state is inaccessible in the crystal, but for completeness sake, is included in Fig. 11 as a grayed-out high energy state labeled “xtal.”

The thermally driven *cis* → *trans* isomerization in a crystal and in solution proceed from different starting states: pB_0_ vs pB_1_. Nevertheless, interconversion from their respective pB state to pG proceeds at similar rates: 3.8 s^−1^ at 288 K in a crystal; 3.6 s^−1^ at 295 K in solution, which implies comparable activation energies for *cis* → *trans* isomerization.

**FIG. 12.**
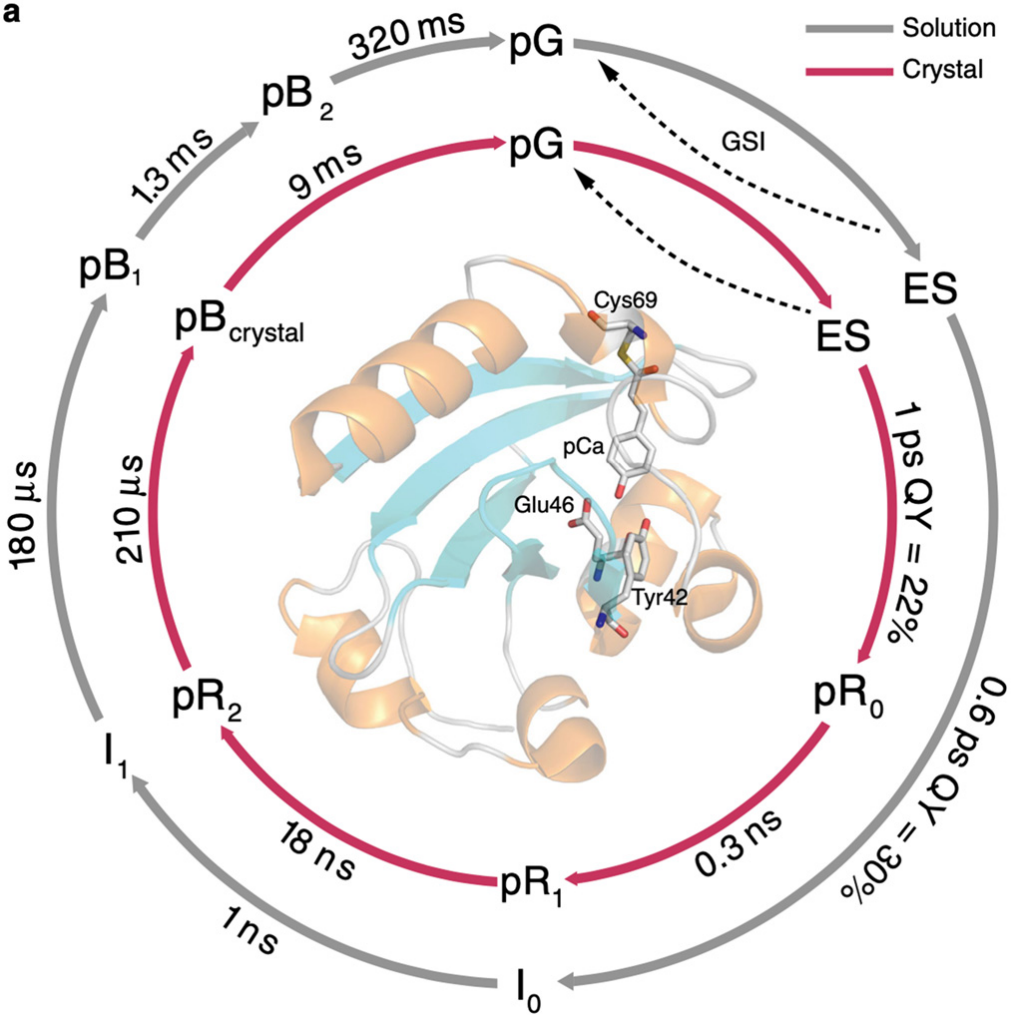
Simplified PYP photocycles deduced from time-resolved visible and mid-IR absorption spectra acquired from solution (gray arrows) and from crushed crystals (red arrows) (Konold *et al.*, 2020). The quantum efficiencies for forming the first *cis* intermediates were extracted from a more complete kinetic scheme. Reprinted with permission from Konold *et al.*, Nat. Chem. **11**(1), 4248 (2020). Copyright 2020 Author(s), licensed under a Creative Commons Attribution (CC BY) license.

A recent time-resolved visible and mid-IR absorption study of PYP in solution and in crystalline form at room temperature with ultrafast time resolution identified intermediates spanning 12 decades of time, from which simplified PYP photocycles were extracted (see Fig. 12) (Konold *et al.*, 2020). Note that these photocycles have a near one-to-one correspondence with that reported by Schotte *et al.* (Schotte *et al.*, 2012). The lifetimes reported for the PYP photocycle in crystalline form, where crystals grown in PEG2000 at pH 6.5 were crushed and sandwiched between parallel windows, are remarkably similar to those reported by Schotte *et al.*, except for the lifetime of the pB_crystal_ state, which is significantly shorter. This difference suggests that PEG2000 interacts with waters of hydration strongly enough to inhibit water penetration and binding to Tyr42/Glu46, thereby speeding recovery to the pG state. The lifetimes reported for the PYP photocycle in solution are very similar to those reported by Cho *et al.*, indicating the spectral features used to characterize the intermediates are sensitive to the same structural changes that affect the SAXS/WAXS patterns. These results provide yet another example of how the protein crystal influence on the PYP photocycle can be rationalized in terms of a restoring force that slows all but the last step in the photocycle.

In summary, the photocycle presented in Fig. 11 is based on five well-defined and DFT-validated structures whose interconversion rates in both crystal and solution environments can be rationalized with a relatively straightforward kinetic scheme in which photoisomerization of pG quickly generates pR_0_, whose shortened distance between the pCA phenolate and sulfur acts like a winch that compacts the protein along that direction and creates strain that is partially relieved in each subsequent transition along the PYP photocycle that leads to the pB_0_ state.

### Influence of precipitant on wild-type and E46Q PYP photocycles in crystals

**FIG. 13.**
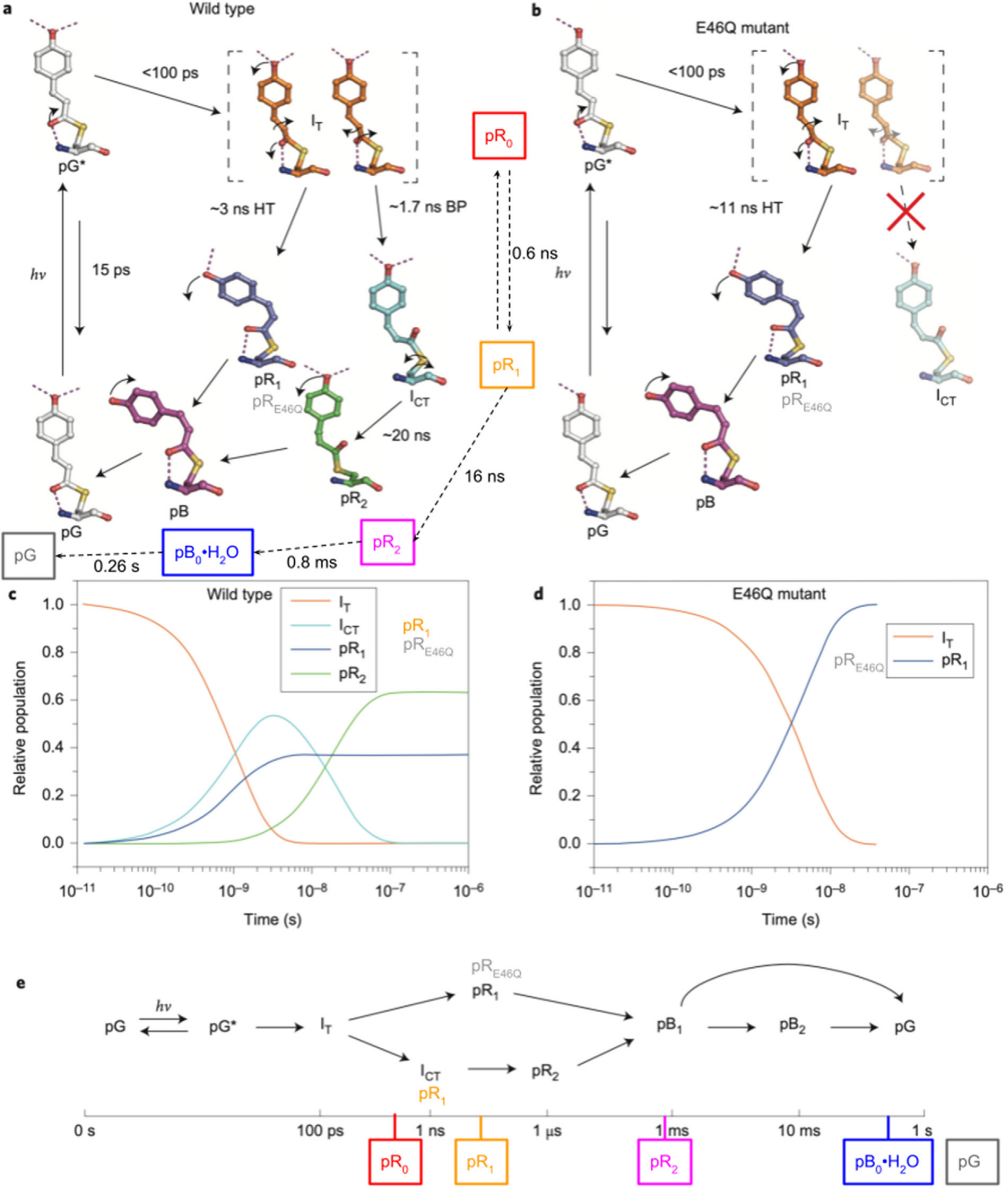
PYP photocycle generated from time-resolved Laue crystallography study of wild-type and E46Q PYP crystals grown with precipitant lacking chloride, as reported by Jung *et al.* (Jung *et al.*, 2013). The top panels depict intermediates characterized in their respective photocycles, the middle panels depict the time-dependent populations of early intermediates, and the bottom panel depicts a timeline with a branching photocycle that accounts for not only multiple pR intermediates that coexist up until their conversion to pB but also to account for non-exponential recovery of the ground, pG state. The color-coded, boxed labels connected with dashed arrows in the upper left panel correspond to intermediates reported by Schotte *et al.* (Schotte *et al.*, 2012). The timeline in the bottom panel is also annotated according to the estimated lifetimes of these intermediates. Reprinted with permission from Jung *et al.*, Nat. Chem. **5**(3), 212–220 (2013). Copyright 2013 Macmillan Publishers Limited.

Following Schotte *et al.* (Schotte *et al.*, 2012), a similar time-resolved crystallography study by Jung *et al.* (Jung *et al.*, 2013) reported PYP photocycles for both wild-type and E46Q PYP, which are shown in Fig. 13 and supplemented with annotation to facilitate comparison with the photocycle reported by Schotte *et al.* Note that in the Schotte photocycle, the integer subscripts for the pR states indicate the order in which they appear; in the Jung photocycles, the intermediate designations have mixed formats. Eleven pump-probe time delays were used to generate the Jung wild-type photocycle, which spanned from 100 ps to 10 ns with four time points per decade and included 100 ns and 1 *μ*s time points. Eleven pump-probe time delays were used to generate the E46Q photocycle, which spanned from 100 ps to 31.6 ns with four time points per decade. In contrast, 42 pump-probe time delays were used to generate the Schotte photocycle, which spanned from 100 ps to 316 ms with four time points per decade, plus −50, 0, and 50 ps time points (spanning nearly 10 decades).

Although I_T_ and pR_0_ both correspond to the first red-shifted *cis* intermediate observed in the PYP photocycle, their refined structures are similar in most respects and their stereochemistry across the C_2_=C_3_ double bond differs significantly. This difference ignited a controversy (Jung *et al.*, 2013; Kaila *et al.*, 2014), but thanks to subsequent studies, the general consensus is that the first *cis* intermediate in the PYP photocycle is best represented by the pR_0_ structure (Pande *et al.*, 2016).

The kinetic equilibrium between pR_0_ and pR_1_ was included in the model reported by Schotte *et al.* because electron density features uniquely associated with pR_0_ persisted beyond where they should when assuming the photocycle proceeds from pR_0_ to pR_1_ irreversibly. This equilibrium, which is supported by DFT calculations of their relative free energies, was not considered in the Jung photocycle, which means the lifetimes found for the early intermediates do not have a simple one-to-one correspondence with the Schotte photocycle. Nonetheless, the state denoted pR_2_, which is common to both Schotte and Jung photocycles, grows with similar time constants. Note that the ∼20 ns time constant reported in the Jung photocycle is approximate due to the lack of pump-probe time delays between 10 and 100 ns.

The intermediate denoted I_CT_ is structurally similar to pR_1_ in the Schotte photocycle, which implies the bicycle-pedal (BP) pathway described by Jung *et al.* is equivalent to the Schotte photocycle. Moreover, the hula-twist (HT) pathway described by Jung *et al.* is equivalent to the photocycle of E46Q PYP, whose sole pR intermediate is structurally very similar to the intermediate denoted pR_1_ in their wild-type photocycle. As this intermediate was denoted pR_E46Q_ in prior studies (Ihee *et al.*, 2005; Tripathi *et al.*, 2012), that designation was added to Fig. 13 to help distinguish this state from the structurally different pR_1_ state reported in the Schotte photocycle.

The structure of the last *cis* intermediate characterized in the Schotte photocycle has a water molecule hydrogen bonded to both Tyr42 and Glu46 and is, therefore, denoted pB_0_•H_2_O. No water was found in the pB structures characterized in prior time-resolved studies of either wild-type or E46Q PYP (Ihee *et al.*, 2005; Rajagopal *et al.*, 2005). Although the pB_0_•H_2_O → pG ground state recovery in the Schotte photocycle occurs with a time constant of approximately 260 ms at 288 K with close to monoexponential kinetics, the pB → pG ground state recovery reported by Schmidt *et al.* was found to be biexponential with the faster process having a time constant of 6 ms at room temperature (Schmidt *et al.*, 2013). Note that the states denoted pB_1_ and pB_2_ were included in the Jung photocycle not because they are distinguishable structurally, but because of the multi-exponential recovery of pG. The branching indicated in their model represents but one of several possible mechanisms that might explain the observed deviation from first-order kinetics.

The Schotte and Jung photocycles differ both early and late in the PYP photocycle. How can these structural and kinetic differences be rationalized?

The time-resolved crystallography study reported by Schotte *et al.* employed crystals grown in D_2_O at pD9 with the precipitant including 2.25M ammonium sulfate and 1.1M NaCl, whereas the other studies employed crystals grown in H_2_O at pH 7 with the precipitant being ∼3M ammonium sulfate. Schmidt *et al.* (Schmidt *et al.*, 2013) pointed out that under their crystallization conditions, two pR intermediates coexist up until pB is formed regardless of whether the precipitant pH was 4, 7, or 9 (Tripathi *et al.*, 2012), which implies pH is likely not the origin of the differences observed. It is doubtful that differences between heavy and normal water could influence the number of intermediates observed. Indeed, a time-resolved Laue crystallography study of PYP grown in H_2_O at pH9 using the same precipitant recipe, i.e., 2.25M ammonium sulfate and 1.1M NaCl, generated electron density maps that were virtually identical with those obtained from crystals grown in D_2_O at pD9, with pB_0_ of both datasets exhibiting water hydrogen bonded to Tyr42/Glu46 at high occupancy (unpublished data). Since the difference in the number of intermediates observed is not due to heavy water or pH, we turn to differences in the precipitant used to generate PYP crystals.

Water being 55.5M, the average number of water molecules available to hydrate the number of ions present with either precipitant recipe is approximately 6, the coordination number expected. Hence, virtually all water in the protein crystal can be characterized as waters of hydration with substantial ion pairing between counterions expected. However, a key difference between these two precipitant recipes is the presence of small monovalent anions and cations when the precipitant includes NaCl. The “*halo*” in the name of the bacterium that produces PYP, *E. halophila*, emphasizes the fact that this organism thrives in the presence of high concentrations of NaCl.

As already explained, a water molecule hydrogen-bonded to Tyr42/Glu46 in the pB_0_•H_2_O structure would be expected to slow the pB_0_•H_2_O → pG transition. Indeed, the pB_0_•H_2_O → pG transition reported by Schotte *et al.* is significantly slower than the pB → pG transition reported in prior studies in which the pB state had no hint of an internal water molecule. If this kinetic difference is indeed due to the presence/absence of a single water molecule hydrogen-bonded to Tyr42/Glu46, the question becomes how might the precipitant influence its occupancy? From a thermodynamic point of view, factors that stabilize water outside the protein will shift the thermodynamic equilibrium toward lower occupancy of water inside the protein. Polyvalent ions hold onto waters of hydration more tightly than monovalent ions. The smaller the ionic radii, the more tightly one would expect it to hold onto water. Moreover, ions capable of directly hydrogen bonding with water, such as the ammonium ion, would be expected to hold onto waters of hydration more strongly than ions without that capability. Hence, we hypothesize that waters of hydration surrounding Cl- are higher in free energy than the water hydrogen bonded to Tyr42/Glu46, which leads to high occupancy water in the crystal pB state. Accordingly, one would expect water to bind to Tyr42/Glu46 in the solution phase pB state whether or not Cl- is present, as most of the water surrounding the protein is “free” and not directly involved in hydrating ions. Hence, one might expect the pB_0_•H_2_O → pG structural transition to proceed at similar rates with a monoexponential time course in the crystal as well as in solution, as was found experimentally. If the presence of chloride is indeed responsible for water penetration in PYP crystals, it would seem prudent to include chloride ions in precipitant recipes used to grow them.

**FIG. 14.**
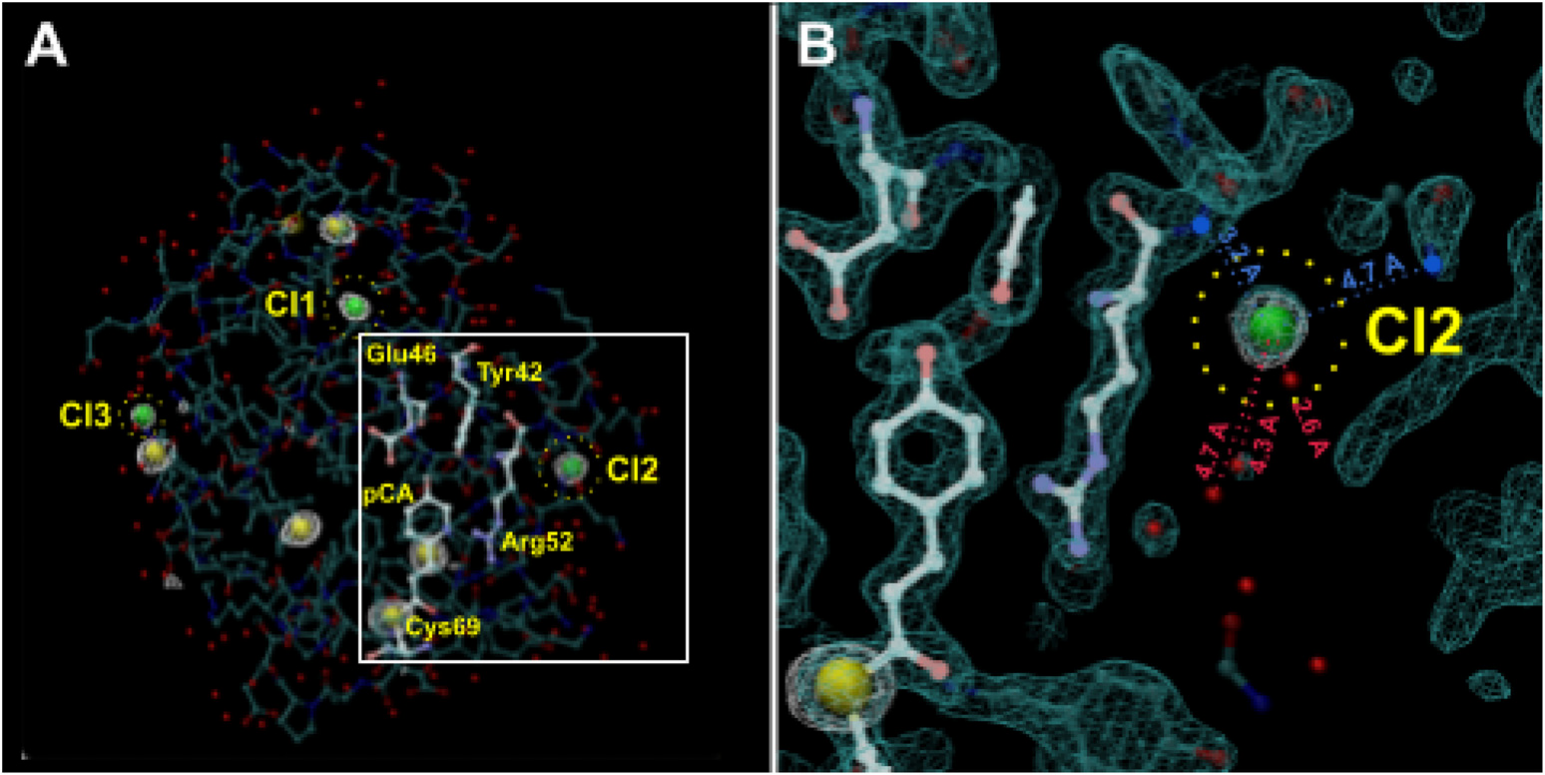
Anomalous scattering of PYP. (a) Anomalous electron density map (*F*^+^-*F*^-^, *φ*_c_-90°) contoured at 3.6 σ in white reveals five internal sulfur (yellow) and three surface chloride (green) sites. (b) Enlarged view of Cl2. Normal density map (2*mF*_o_-*DF*_c_, *φ*_c_) contoured at 1.6 σ in cyan, anomalous density (*F*^+^-*F*^-^, *φ*_c_-90°) contoured at 3.6 σ in white. Green balls: Cl^-^ ions; small red balls: waters of hydration with distances from Cl2; blue balls: positive charged amino groups exposed to the surface with distances from Cl2 (PDB ID 9AZ7).

If the presence or absence of chloride ions in the precipitant recipe can affect the occupancy of water in the pB_0_ state, might they also affect the number of pR intermediates detectable in the PYP photocycle? To investigate this possibility, we acquired diffraction datasets at an x-ray energy of 8.04 keV from crystals grown in ammonium sulfate with and without NaCl (supplementary material, Table 1) and identified eight sites that exhibited anomalous scattering (Fig. 14). Five were found inside the protein, which correspond to five of six sulfurs in PYP (the N-terminus Met has a weak signal), and three were found outside the protein at sites that were previously identified as crystallographic waters (PDB ID 9AZ7). No anomalous scattering was observed in these three sites when NaCl was dialyzed out from the crystal (PDB ID 9AZ9). When the structure was refined with chloride in those sites, the occupancies were found to be 0.82, 0.80, and 0.73 for Cl1, Cl2, and Cl3, respectively. One of those chlorides, Cl2, is located near Arg52 and in a region identified as a crystal contact. We hypothesize that a chloride ion in this site destabilizes the pR_E46Q_ structure found in the Jung *et al.* photocycles for wild-type and E46Q PYP and accounts for the fact that pR_E46Q_ is absent in the Schotte photocycle.

## FEMTOSECOND TIME-RESOLVED X-RAY STUDIES

**FIG. 15.**
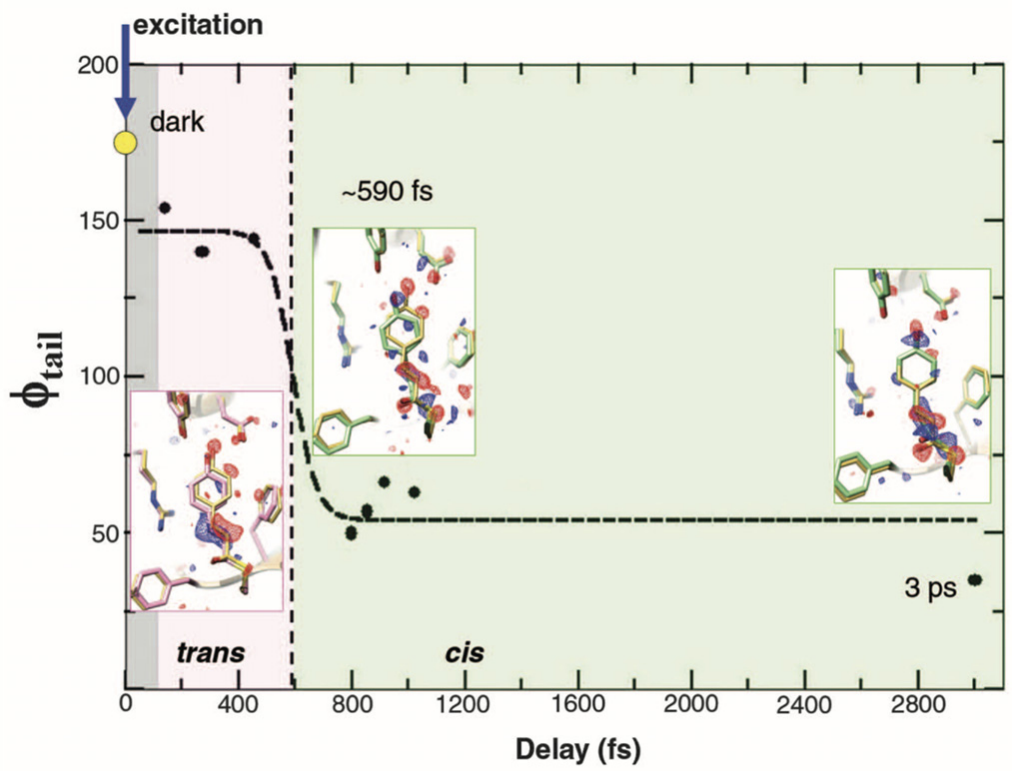
Chromophore tail torsional angle dynamics. Torsional angle φ_tail_ (solid spheres) is from structural refinement at various delays. Dashed line: fit to empirical function used to deduce the transition time (∼590 fs). Gray region: not time-resolved. Pink region: twisted *trans* on excited state potential energy surface. Light green region: *cis* on ground state potential energy surface. Insets: structures of PYP_fast_ (pink), PYP_slow_, and PYP_3ps_ (light green), and dark structure PYP_ref_ in yellow. Difference electron density is shown in red (–3σ) and blue (3σ). Reprinted with permission from Pande *et al.* Science **352**(6286), 725–729 (2016). Copyright 2016 American Association for the Advancement of Science.

The advent of the x-ray free electron laser, first demonstrated at the LCLS, created a unique opportunity to acquire crystal structures of PYP with sub-picosecond time resolution. This effort presented many challenges, but the *tour de force* efforts by a small army of scientists with diverse expertise managed to extract structures of transient intermediates from about a half million single-shot diffraction images acquired following flash photoactivation of micrometer-sized crystals (Pande *et al.*, 2016). Briefly, PYP crystals in the 1–5 *μ*m range, whose size distribution was skewed toward smaller sizes, were delivered through a nozzle into a vacuum chamber where they were photoactivated with ∼140 fs, 450 nm laser pulses at a power density of 0.8 mJ mm^−2^ and subsequently probed with 40 fs, 9.5 keV, 2 mJ x-ray pulses focused to 1 *μ*m, whose time of arrival was controlled and recorded shot by shot at a 120 Hz repetition rate. The laser penetration depth at this wavelength was estimated to be about 3 *μ*m, which implies fairly uniform photoactivation throughout the crystal. Atomic structures shown in Fig. 15 were refined against difference electron density maps generated from 1.6 Å resolution diffraction images acquired without laser, PYP_dark_ (82 214 images), and from images acquired over three specified ranges of pump-probe delays: PYP_fast_ (0.1–0.4 ps; 81 327 images), PYP_slow_ (0.8–1.2 ps; 157 082 images), and PYP_3ps_ (∼3 ps; 76 411 images). R_free_ for the time-resolved structures ranged from 24 to 26.

The progress along the photoisomerization reaction coordinate is best described by the dihedral angle across the C_2_=C_3_ double bond in pCA, φ_tail_, which is shown in Fig. 15 along with inset charts of refined structures. In the dark state, φ_tail_ was reported to be 172°, which differs modestly from the 180° angle expected when free of external influences. Hence, the surrounding protein side chains impose a modest amount of torsional strain on this coordinate. This strain breaks the symmetry of the three-state potential shown in Fig. 7 and introduces a steric bias that can steer the reaction pathway to the left or right. Upon breaking the C_2_=C_3_ double bond, one would expect *φ_t_*_ail_ to continue in that direction and come to a new equilibrium orientation in which that strain is relieved. Indeed, after photoexcitation of pCA, *φ*_tail_ evolves from 172° to 146°^‡^, which is still far from the avoided crossing near 90°, demonstrating that the pCA remains in its excited electronic state. Upon accessing the avoided crossing and transitioning from the excited to the ground electronic state, pCA will either return to the 172° *trans* state (pG) or continue toward the 33° *cis* state (pR_0_). To follow this trajectory, *φ*_tail_ was estimated from binned sets of approximately 40 000 indexed images according to their timestamps and shown as black dots in Fig. 15. The transition time reported for accessing the *cis* ground state was estimated from these data to be ∼590 fs. Refinement was not successful with binned images centered at 699 fs, which the authors presumed was due to the existence of a mixture with some molecules relaxing toward *cis* and others back toward *trans*. The depiction of the φ_tail_ trajectory as a rapid transition from *trans* to *cis* after an induction period is deceptive and discordant with expectations. The first excited state in the three-state potential has a small activation barrier that separates the excited state structure from the reactive region of the excited state potential. According to transition state theory, one expects first order kinetics for crossing that barrier and, therefore, represents a rate-limiting step. Once over that barrier, the trajectory proceeds along a steep slope and either returns to the *trans* (high probability) or proceeds to the *cis* (low probability) side of the potential, a process that likely requires only a few tens of femtoseconds. Since the trajectory along the reactive portion of the electronic potential is fast compared to the rate at which it is accessed, one cannot experimentally capture the intermediate during passage through the conical intersection. The best one can hope for is to characterize the relative ground and excited state populations and refine structures of both states as a function of time. Nevertheless, the new and important experimental details generated by this study can inform QM/MM (quantum mechanics/molecular mechanics) calculations, from which atomic level structural details can, in principle, be unveiled.

^‡^The 136° value reported in Table I of Pande *et al.* appears to be a typo, as the average of the “fast” binned results reported in the SI is 146°, which agrees with the points plotted in Fig. 15.

Tracking structural changes in PYP on the sub-picosecond time scale is very challenging owing in part to the relatively low single-photon quantum efficiency for *trans*-to-*cis* isomerization, which is typically <20%. The temptation is to raise the laser fluence to maximize the probability for inducing structural change. Unfortunately, when photoactivating PYP with femtosecond laser pulses, the threshold for nonlinear absorption is crossed before the laser fluence is sufficient to photoactivate most of the pCA chromophores in the crystal. Since the onset of nonlinear absorption typically scales as the square of the peak power, raising the power above this threshold can trigger irreversible structural changes and decrease the yield of the *cis* state. Indeed, Hutchison *et al.* (Hutchison *et al.*, 2016) estimated the single-shot yield of the long-lived pB states in PYP as a function of laser energy density with femtosecond pulses and found a peak at 0.3 mJ mm^−2^ followed by a sharp decrease with increasing power density, owing to the onset of nonlinear absorption. The power density used by Pande *et al.* is several times higher than that recommended by Hutchison, which implies some of the structural changes observed may be affected by processes other than *trans*-to-*cis* isomerization. At this power density and with the pump-probe delay set to 3 ps, Pande *et al.* estimated the *cis* yield be 10.1%, which is modestly lower than the 14% yield estimated by Lincoln *et al.* when photoactivating PYP in solution under similar laser conditions (Lincoln *et al.*, 2012)

**FIG. 16.**
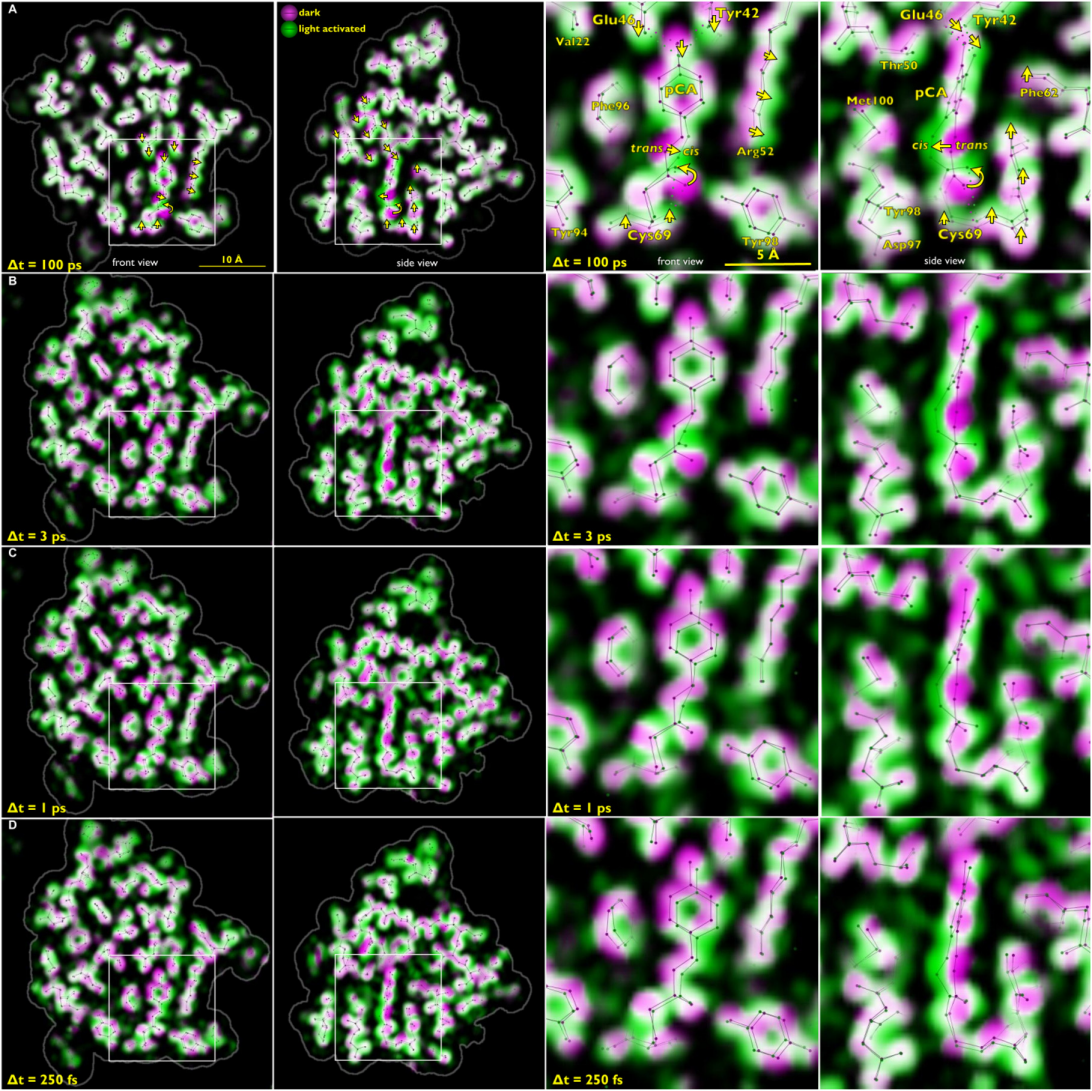
Front and side views of time-resolved structural changes recorded as a function of time delay after photoexcitation of PYP. The right two panels show an expanded, annotated view of the boxed region in the left two panels. The ground state electron density map in the color-coded overlays is colored magenta, and the time-resolved map is colored green. Where magenta and green overlap, the electron density blends to white. The magenta-to-green color gradient unveils the direction of atomic motion. Large-amplitude displacements in (a) are indicated with yellow arrows. The stick models correspond to refined structures for the ground state (pG) and the time-resolved intermediate. Hydrogen bonds to the pCA chromophore are indicated as dotted lines in the right side of panel (a). Row (a): PDB ID: 4B9O (Schotte *et al.*, 2012); Row (b)–(d): PDB IDs: 5HDS, 5HDD, 5HDC (Pande *et al.*, 2016). Magenta: *F*_c_ map, Green *F*_c_ + Δ*F*_o_, map. Rendered with LaueMap.

When visualizing structural change using weighted difference maps contoured at a specified sigma level, as employed in Fig. 15, only changes that rise above that sigma level show up, and it is often difficult to interpret the origin of extraneous positive and negative blobs of electron density that pop up between atoms. A more informative way to visualize structural changes in proteins employs color-coded overlays of electron density maps generated for the dark (magenta) and light (green) states. Where overlapped, these colors blend to white. Atom displacement is unveiled by a magenta to green color gradient that corresponds to the direction of atomic motion. This method of rendering was employed in Fig. 16, in which the 3 ps (PYP_3ps_), 1 ps (PYP_slow_), and 250 fs (PYP_fast_) structures are shown along with corresponding data acquired at 100 ps by Schotte *et al.* With high S/N data, the magenta coloration on one side of a displaced atom has approximately the same boldness as the green coloration on the opposite site, with bolder colors corresponding to larger displacement. This rendering scheme also helps unveil correlated motion of adjacent atoms. For example, formation of the *cis* isomer shortens the overall length of the pCA chromophore and pulls the Tyr42 and Glu46 downward. This motion shows up quite clearly in the 100 ps map. Moreover, in response to photoisomerization, atoms in the Arg52 and Thr50 side chains exhibit collective motion in a well-defined direction. The similarity of the magenta-green color gradients exhibited around the pCA chromophore in the 1, 3, and 100 ps maps suggests that the pR_0_ structure, as defined by the 100 ps map, indeed represents the first *cis* intermediate in the PYP photocycle. Moreover, the pCA geometry at 3 ps in Table I of Pande *et al.* is remarkably similar to the 100 ps pR_0_ structure, but not the I_T_ structure proposed by Jung and Schmidt, and should put to rest the controversy (Kaila *et al.*, 2014) over which structure, I_T_ or pR_0_, best describes the first *cis* intermediate in the PYP photocycle. The 250 fs, 1 ps, and 3 ps maps exhibit more extraneous green coloration between atoms than the 100 ps map, which indicates poorer S/N. The magenta-green color gradients exhibited by side chains surrounding pCA in the 1, 3, and 100 ps maps exhibit both similarities and differences, which likely reflects the overall protein structural relaxation needed to accommodate the sudden local structural change occurring when pCA isomerizes, as well as the vibrational cooling that ensues following the large temperature jump experienced by pCA when transitioning from the excited to the ground state, an event that releases of the order of 20 000 cm^−1^ energy.

The 250 fs structure in panel (d) of Fig. 16 corresponds to PYP_fast_, which includes time points spanning 0.1–0.4 ps, and should be dominated by structural changes that arise upon photoexcitation of PYP, not photoisomerization. Moreover, the excited state population in the crystal would be expected to be more than five times greater than the population of *cis* generated when relaxing back to the ground state and should, therefore, generate much higher S/N maps than those depicting the ground state *cis* isomer. The fact that the magenta to green color gradients surrounding the pCA chromophore in PYP_fast_ have striking similarities to those observed at 1 and 3 ps suggests that this map is contaminated by a *cis* conformer, which would need to be properly subtracted to characterize the true structure of the excited state. It is perhaps worth noting that structural changes are evident throughout the protein in the 250 fs structure. If we assume structural change can propagate from the epicenter at a rate comparable to the speed of sound in water, 1500 m s^−1^, then one would expect the magenta to green gradients in the maps of panel (d) to be confined within 6 Å from the pCA chromophore. The fact that color gradients are observed well beyond this distance and throughout the protein suggests that the S/N of the difference electron density maps is poor, and structures refined against these difference maps may be distorted from the true structures.

It is perhaps worth noting that the precipitant used to grow crystals for the femtosecond time-resolved study was 3.2 to 3.3M sodium malonate, which represents yet another precipitant recipe. After growth, the crystals were filtered through a 10 *μ*m stainless steel mesh and resuspended in 2.8M sodium malonate at pH 7. The 200 ns structure, denoted PYP_200ns_, depicts a mixture of pR states, namely, pR_E46Q_ and pR_2_, as found when PYP crystals were grown in 3M ammonium sulfate. At pH 7, the malonate anion has a −2 charge, similar to sulfate. The presence of pR_E46Q_ in PYP crystals grown in a precipitant that contains an abundance of sodium cations, but no chloride anions, adds further evidence to our hypothesis that chloride might destabilize the pR_E46Q_ intermediate so only pR_2_ is represented when the precipitant recipe includes chloride.

Serial crystallography studies that involve sample injection into vacuum require a very large number of indexable diffraction images to refine a single structure, which is in large part due to the partiality problem. Consequently, the number of time points that could be acquired during LCLS beamtime allocated to this project was limited. Since reaction rates are temperature dependent, one would like to know, and better yet, control the crystal temperature. The temperature of the crystals in this study before photoactivation is unreported, but is well below ambient temperature due to evaporative cooling that ensues upon injection into vacuum. The magnitude of the cooling would be expected to be crystal size dependent with crystals having a larger surface area to volume ratio, i.e., smaller crystals, cooled to a lower temperature. Hence, a distribution of temperatures is represented in the diffraction images. As discussed earlier, the temperature of the pCA chromophore and its immediate surroundings are elevated relative to the surrounding protein immediately after photoexcitation, and when transitioning to the *cis* state, a significant amount of potential energy is released, which elevates the temperature of the pCA chromophore much further, surely enlarging the B factors of its atoms and those immediately surrounding it, but would be expected to thermally equilibrate with the surrounding protein within a few picoseconds. This thermal history further complicates the interpretation of the sub-picosecond time-resolved maps.

Although synchrotron-based time-resolved crystallography studies lack ultrafast time resolution, they afford numerous advantages such as the ability to precisely control sample temperature, to determine diffraction partiality via repeated exposures of the same crystal and to achieve high level photoactivation of crystals without interference from nonlinear absorption. For example, Schotte *et al.* (Schotte *et al.*, 2012) stretched the laser pulses to 125 ps and boosted the power density to 3 mJ mm^−2^, several times higher than used by Pande *et al.*, which proved sufficient to excite most of the pCA chromophores in the laser illumined volume of the crystal multiple times without suffering the ill effects of nonlinear absorption. This approach improved the S/N of the pump-induced differences observed in the diffraction data, boosted the quality of the maps generated from those data, and allowed for the acquisition of 42 time points in about 6 h of data acquisition time. From those data, four high quality structures, pR_0_, pR_1_, pR_2_, and pB_0_, as well as their interconversion kinetics could be determined (Schotte *et al.*, 2012). For comparison, Pande *et al.* acquired 10.3 × 10^6^ diffraction images within 45 h of data acquisition that spanned five 12 h shifts, of which 9.4 × 10^5^ were hits and 6 × 10^5^ could be indexed. Each shift was typically setup for one of several nominal time delay settings that included −1 ps, 300 fs, 900 fs, 3 ps, and 200 ns. Structures were determined at five nominal time points, where PYP_fast_ (0.1–0.4 ps) and PYP_slow_ (0.8–1.2 ps) were determined from a subset of images acquired with the nominal delay set to 300 and 900 fs, respectively. When comparing the 100 ps Schotte *et al.* time point with the Pande *et al.* time points in Fig. 16, the extraneous green coloration in the magenta-green maps is significantly worse in the LCLS maps, indicating poorer S/N electron density maps. Nevertheless, the heroic efforts by this team to characterize the structure of PYP on sub-picosecond time scales have produced significant new insight into the early dynamics of PYP photoisomerization.

Finally, though the transition time from the excited state to the first *cis* intermediate from crystallography and spectroscopy are in good agreement, ∼0.6 ps for crystallography and 0.6–1 ps from spectroscopy (Lincoln *et al.*, 2012), it should be recognized that both determinations are model dependent. Nevertheless, whether as fast as 0.6 ps or as slow as 2.4 ps, the first *cis* intermediate is formed on a similar time scale as vibrational cooling and generates the highly contorted pR_0_ intermediate, the first in a series of well-characterized structures that ultimately lead to the PYP signaling state (Schotte, 2012).

## CONCLUSIONS

Over the nearly 40 years since its discovery, PYP has arguably become one of the most thoroughly investigated proteins found in nature. We know its high resolution static structure, how its photocycle is influenced by numerous external factors, how heterogeneity via conformational substates can affect reaction rates and pathways, and how its structure evolves over 12 decades of time with sub-picosecond time resolution, all of which allows biophysicists to literally watch a signaling protein as it functions. The first *cis* intermediate, which is formed within 2.4 ps and perhaps as fast as 0.6 ps, is highly contorted and well described by the pR_0_ structure reported by Schotte *et al.* (Schotte *et al.*, 2012). Persistent strain in the pCA chromophore drives subsequent structural transitions that ultimately lead to the signaling state. The PYP photocycle in crystals is influenced by the composition of the precipitant with differences observed both early and late in the photocycle. Nevertheless, the PYP photocycle in crystals whose precipitant includes chloride was found to be kinetically similar to the PYP photocycle in solution, with the modest differences observed readily rationalized in terms of the restoring forces imposed on PYP by crystal contacts. Compared to rhodopsin and bacteriorhodopsin, the single-photon quantum efficiency for photoisomerization in PYP is quite low, which may account for the relatively large concentration of PYP produced in *E. halophila*. The final chapter has not yet been written, as we still do not know what its signaling partner is, nor how unfurling the 25-residue N-terminal domain presumably facilitates recognition by that partner. As this process occurs on the millisecond time scale, the emphasis of future studies will likely shift from the ultrafast to more pedestrian time scales in the realm of diffusion and reversible binding.

## SUPPLEMENTARY MATERIAL

See the supplementary material for Table I.

## Data Availability

The data that support the findings of this study are available from the corresponding author upon reasonable request.
